# Multi-sample $\zeta$-mixup: richer, more realistic synthetic samples from a *p*-series interpolant

**DOI:** 10.1186/s40537-024-00898-6

**Published:** 2024-03-23

**Authors:** Kumar Abhishek, Colin J. Brown, Ghassan Hamarneh

**Affiliations:** 1 School of Computing Science, Simon Fraser University, 8888 University Drive, Burnaby, V5A 1S6 Canada; 2 Engineering, Hinge Health, 455 Market Street, Suite 700, San Francisco, 94105 USA

**Keywords:** Deep learning, Classification, Data augmentation, Mixup, Intrinsic dimensionality, Data manifold

## Abstract

Modern deep learning training procedures rely on model regularization techniques such as data augmentation methods, which generate training samples that increase the diversity of data and richness of label information. A popular recent method, *mixup*, uses convex combinations of pairs of original samples to generate new samples. However, as we show in our experiments, *mixup*  can produce undesirable synthetic samples, where the data is sampled off the manifold and can contain incorrect labels. We propose $\zeta$-*mixup*, a generalization of *mixup*  with provably and demonstrably desirable properties that allows convex combinations of ${T} \ge 2$ samples, leading to more realistic and diverse outputs that incorporate information from ${T}$ original samples by using a *p*-series interpolant. We show that, compared to *mixup*, $\zeta$-*mixup*  better preserves the intrinsic dimensionality of the original datasets, which is a desirable property for training generalizable models. Furthermore, we show that our implementation of $\zeta$-*mixup*  is faster than *mixup*, and extensive evaluation on controlled synthetic and 26 diverse real-world natural and medical image classification datasets shows that $\zeta$-*mixup*  outperforms *mixup*, CutMix, and traditional data augmentation techniques. The code will be released at https://github.com/kakumarabhishek/zeta-mixup.

## Introduction

Deep learning-based techniques have demonstrated unprecedented performance improvements over the last decade in a wide range of tasks, including but not limited to image classification, segmentation, and detection, speech recognition, natural language processing, and graph processing [1–4]. These deep neural networks (DNNs) have a large number of parameters, often in the tens to hundreds of millions, and training accurate, robust, and generalizable models has largely been possible because of large public datasets [5–7], efficient training methods [8, 9], hardware-accelerated training [10–13], advances in network architecture design [14–16], advanced optimizers [17–20], new regularization layers [21, 22], and other novel regularization techniques. While techniques such as weight decay [23], dropout [21], batch normalization [22], and stochastic depth [24] can be considered as “data independent” regularization schemes [25], popular “data dependent” regularization approaches include data augmentation [14, 26–29] and adversarial training [30, 31].

Given the large parameter space of deep learning models, training on small datasets tends to cause the models to overfit to the training samples. This is especially a problem when training with data from high-dimensional input spaces, such as images, because the sampling density is exponentially proportional to $1/{\mathcal {D}}$, where ${\mathcal {D}}$ is the dimensionality of the input space [32]. As ${\mathcal {D}}$ grows larger (typically $10^4$ to $10^6$ for most real-world image datasets), we need to increase the number of samples exponentially in order to retain the same sampling density. As a result, it is imperative that the training datasets for these models have a sufficiently large number of samples in order to prevent overfitting. Moreover, deep learning models generally exhibit good generalization performance when evaluated on samples that come from a distribution similar to the training samples’ distribution. In addition to their regularization effects to prevent overfitting [33, 34], data augmentation techniques also help the training by synthesizing more samples in order to better learn the training distributions.

Traditional image data augmentation techniques include geometric- and intensity-based transformations, such as affine transformations, rotation, scaling, zooming, cropping, adding noise, etc., and are quite popular in the deep learning literature. For a comprehensive review of data augmentation techniques for deep learning methods on images, we refer the interested readers to the survey by Shorten et al. [35]. In this paper, we focus on a recent and popular data augmentation technique based on a rather simple idea, which generates a convex combination of a pair of input samples, variations of which are presented as *mixup* [36], Between-Class learning [37], and SamplePairing [38]. The most popular of these approaches, *mixup* [36], performs data augmentation by generating new training samples from convex combinations of pairs of original samples and linear interpolations of their corresponding labels, leading to new training samples, which are obtained by essentially overlaying 2 images with different transparencies, and new training labels, which are soft probabilistic labels. Other related augmentation methods can broadly be grouped into 3 categories: (a) methods that crop or mask region(s) of the original input image followed by *mixup*  like blending, e.g.,  CutMix [39] and GridMix [40], (b) methods that generate convex combinations in the learned feature space, e.g.,  *manifold mixup* [41] and MixFeat [42], and (c) methods that add a learnable component to *mixup*, e.g.,  AdaMixUp [25], AutoMix [43], and AutoMix [44]. A comparison of existing mixing-based data augmentation methods is presented in Table 2.

**Fig. 1:**
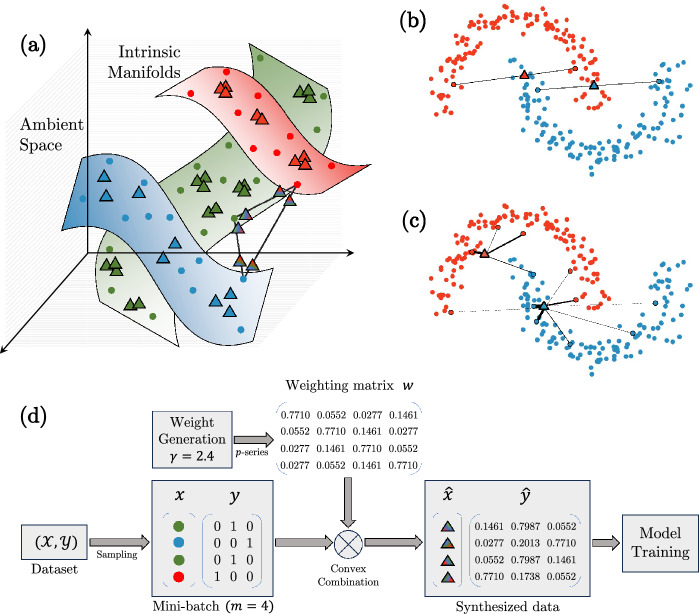
Overview of *mixup*  (**b**) and $\zeta$-*mixup*  (**a**, **c**, **d**). The original and synthesized samples are denoted by $\circ$ and $\bigtriangleup$ respectively, and line segments indicate which original samples were used to create the new ones. The line thicknesses denote the relative weights assigned to original samples. Observe how $\zeta$-*mixup*  can mix any number of samples (e.g.,  3 in (**a**), 4 or 8 in (**c**), and 4 in (**d**)), and that $\zeta$-*mixup* ’s formulation allows the generated samples to be close to the original distribution while still incorporating rich information from several samples. **d** Illustrates a toy dataset with 3 classes, wherein a mini-batch of 4 elements is sampled, then the data and the labels are mixed using a set of weights generated with an example value of the hyperparameter $\gamma$, and finally this synthesized data is used to train a classification model

**Fig. 2:**
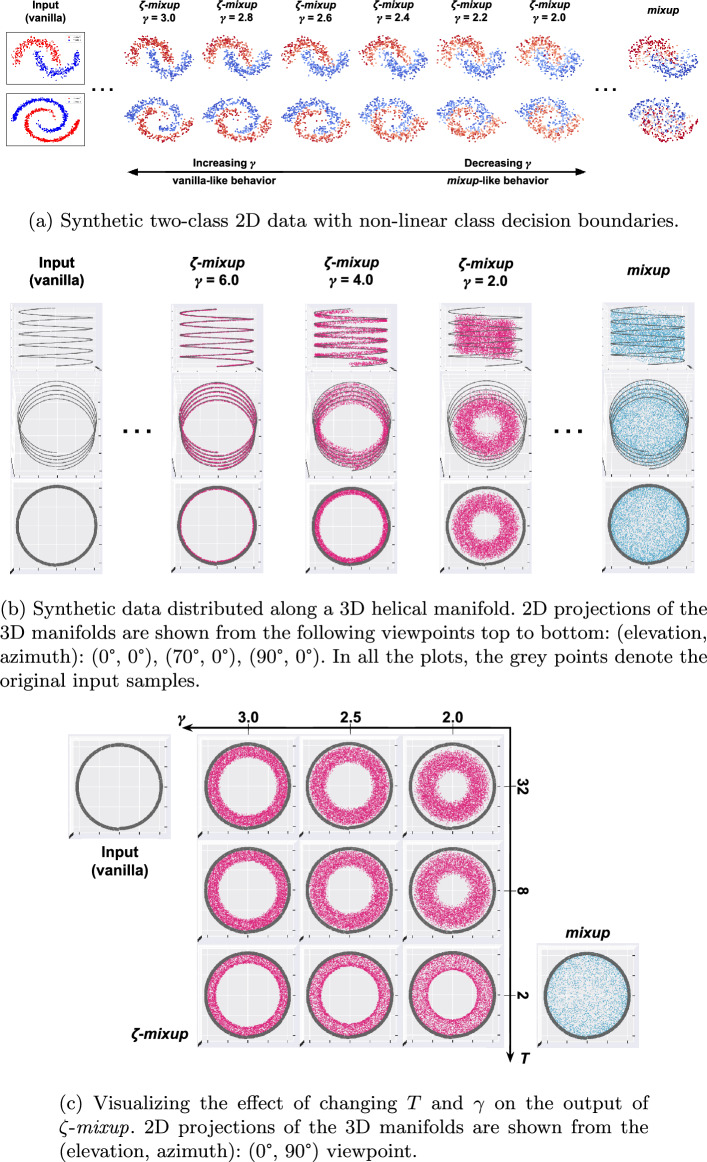
Visualizing how *mixup*  and $\zeta$-*mixup*  synthesize new samples. Notice that *mixup*  produces samples that (**a**) are assigned wrong labels and (**b**, **c**) are sampled off the original data manifold, with an extreme example being where the points are sampled from the hollow region in the helix. A moderately low value of $\gamma$ allows for a more reasonable exploration of the data manifold, with higher values of ${T}$ allowing for more diversity in the synthesized points

**Fig. 3:**
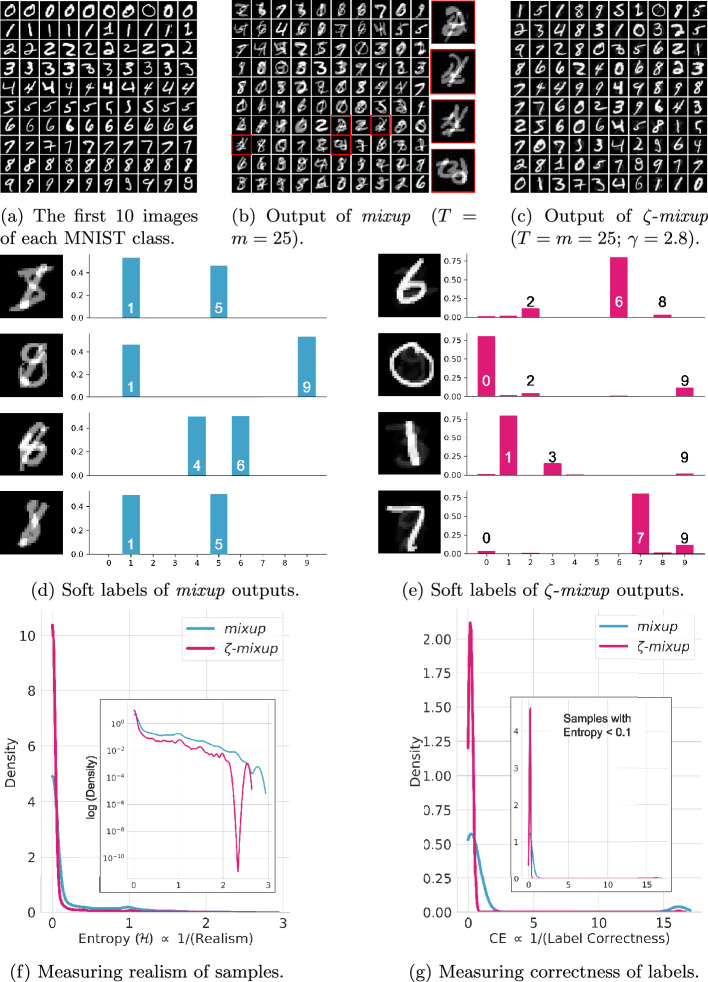
Visualizing the results obtained using *mixup*  (**b**) and $\zeta$-*mixup*  (**c**) on images (**a**) from the MNIST dataset. In **d** and **e**, we visualize the probabilistic “soft” labels assigned to images generated by *mixup*  and $\zeta$-*mixup*  respectively. Notice how all images in **d** look close to the digit “8” while their assigned soft labels do not contain the class “8”. $\zeta$-*mixup*  alleviates this issue and the soft labels in **e** correspond exactly to the class the synthesized images belong to. Also note how *mixup*  produces images with a wrong label, i.e., a label different from the original labels of the two images it is interpolated from. In **f** and **g**, we evaluate the realism of *mixup* ’s and $\zeta$-*mixup* ’s generated samples and the correctness of the corresponding labels by measuring the entropy of the Oracle’s predictions (${\mathcal {H}}$) and the cross entropy of the Oracle’s predictions with the soft labels (CE) respectively. For both **f** and **g**, lower values are better

**Fig. 4:**
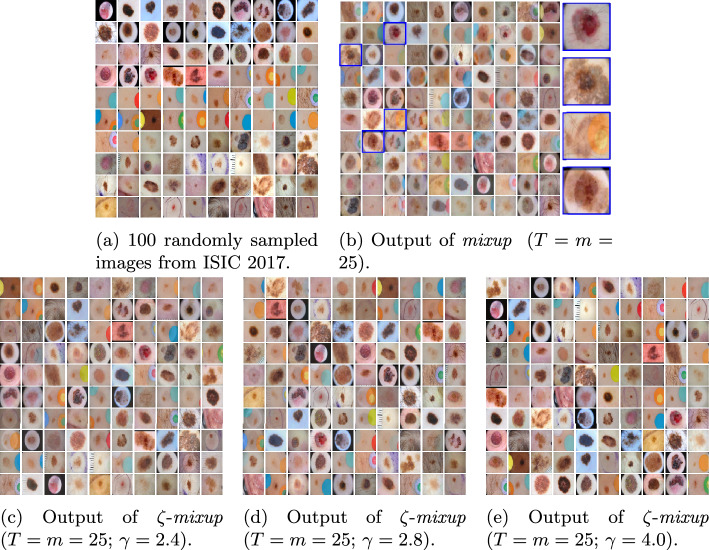
Visualizing the results obtained using *mixup*  (**b**) and $\zeta$-*mixup*  (**c**, **d**, **e**) on images (**a**) from the ISIC 2017 dataset, with three values of $\gamma \ (2.4, 2.8, 4.0)$ used for $\zeta$-*mixup*. Note how *mixup*  synthesizes unrealistic images with ghosting (selected images highlighted in blue in **b**), as evidenced by either multiple lesions overlapping or with artifacts (dark corners, stickers, ink markers) overlapping the lesion. On the other hand, for all values of $\gamma$, $\zeta$-*mixup*  produces visibly more realistic images

*mixup*, however, can lead to ghosting artifacts in the synthesized samples (as we show later in the paper, e.g.,  in Fig. 3), in addition to generating synthetic samples with wrong class labels. Moreover, because *mixup*  uses a convex combination of only a pair of points, it can lead to the synthetic samples being generated off the original data manifold (Fig. 1a). This in turn leads to an inflation of the manifold, which can be quantified by an increase in the intrinsic dimensionality of the resulting data distribution, as shown in Fig. 6, which is undesirable since it has been shown that deep models trained on datasets with lower dimensionalities generalize better to unseen samples [45]. Additionally, *mixup*-like approaches, which crop or mask regions of the input images, may degrade the training data quality by occluding informative and discriminatory regions of images, which is highly undesirable for high-stakes applications such as medical image analysis tasks.

The primary hypothesis of *mixup*  and many of its derivatives is that a model should behave linearly between any two training samples, even if the distance between samples is large. This implies that we may train the model with synthetic samples that have very low confidence of realism; in effect over-regularizing. We instead argue that a model should only behave linearly nearby training samples and that we should thus only generate synthetic examples with high confidence of realism. This is supported by research in cognitive sciences for humans’ categorical perception, where it has been shown that human perception between object category boundaries is warped and is not as linear as *mixup*  seems to suggest [46–49]. To achieve this, we propose $\zeta$-*mixup*, a generalization of *mixup*  with provably desirable properties that addresses the shortcomings of *mixup*. $\zeta$-*mixup*  generates new training samples by using a convex combination of ${T}$ samples in a training batch, requires no custom layers or special training procedures to employ, and is faster than *mixup*  in terms of wall-clock time. We show how, as compared to *mixup*, the $\zeta$-*mixup*  formulation allows for generating more realistic and more diverse samples that better conform to the data manifold (Fig. 1b) with richer labels that incorporate information from multiple classes, and that *mixup*  is indeed a special case of $\zeta$-*mixup*. We show qualitatively and quantitatively on synthetic and real-world datasets that $\zeta$-*mixup* ’s output better preserves the intrinsic dimensionality of the data than that of *mixup*. Finally, we demonstrate the efficacy of $\zeta$-*mixup*  on 26 datasets comprising a wide variety of tasks from natural image classification to diagnosis with several medical imaging modalities.

**Fig. 5:**
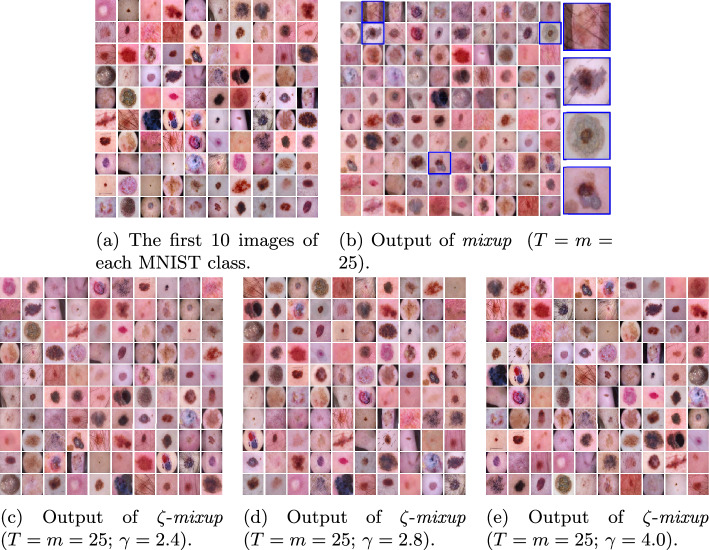
Visualizing the results obtained using *mixup*  (**b**) and $\zeta$-*mixup*  (**c**, **d**, **e**) on images (**a**) from the ISIC 2018 dataset, with three values of $\gamma \ (2.4, 2.8, 4.0)$ used for $\zeta$-*mixup*. Similar to Fig. 4, *mixup*  synthesizes unrealistic images with ghosting (selected images highlighted in blue in **b**), with multiple lesions overlapping, with artifacts (hair) overlapping the lesion, or with unrealistic anatomical arrangements (lesion, hair overflowing outside the body). And as before, for all values of $\gamma$, $\zeta$-*mixup*  produces more realistic images

**Fig. 6:**
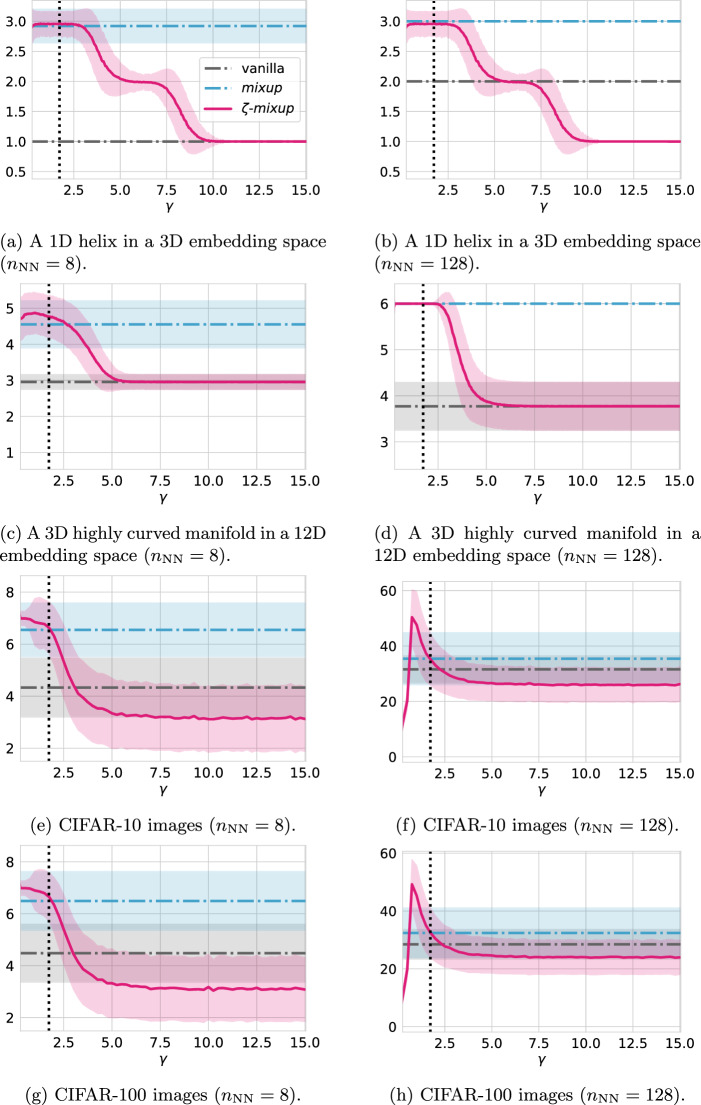
Visualizing how $\zeta$-*mixup*  affects the local intrinsic dimensionality of synthesized datasets distributed as 1D helices (**a**, **b**) and 3D manifold (**c**) in a higher dimensional embedding space as the hyperparameter $\gamma$ changes. The mean and the standard deviation of the intrinsic dimensionality are shown using lines (bold or dashed-dotted) and shaded bands respectively. The vertical dotted line in all the plots denotes the value of $\gamma =\gamma _{\textrm{min}}$ (Theorem 1)

**Fig. 7:**
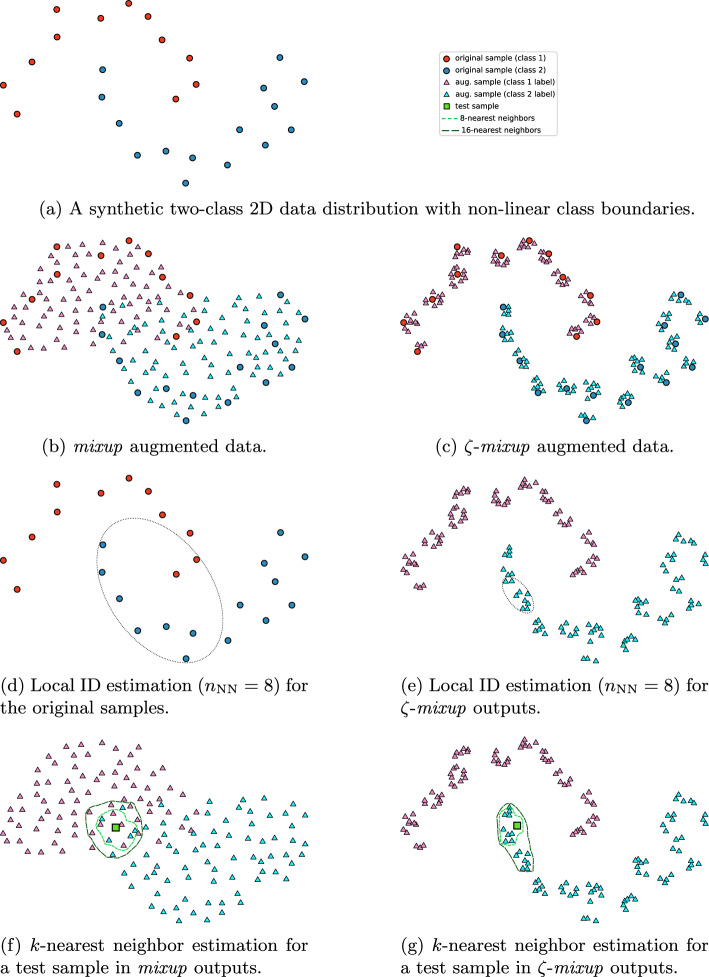
Augmenting a 2D data distribution with non-linear class boundaries (**a**) with *mixup* (**b**) and $\zeta$-*mixup* (**c**). Notice how $\zeta$-*mixup* generates samples closer to the original data, and this explains why the local intrinsic dimensionality (ID) estimates for $\zeta$-*mixup* (**d**) may sometimes be lower than the original dataset (**e**) (Fig. 6): the Fukunaga-Olsen method for local ID estimation using PCA based on nearest-neighbor sampling may yield a more compact distribution for $\zeta$-*mixup*. Conversely, with *mixup*, a test sample may lie in the vicinity (calculated using *k*-nearest neighbors; $k = \{8, 16\}$) of training samples from classes different from the test image’s correct label, leading to an incorrect prediction (**f**). This is less likely with $\zeta$-*mixup* (**g**)

**Fig. 8:**
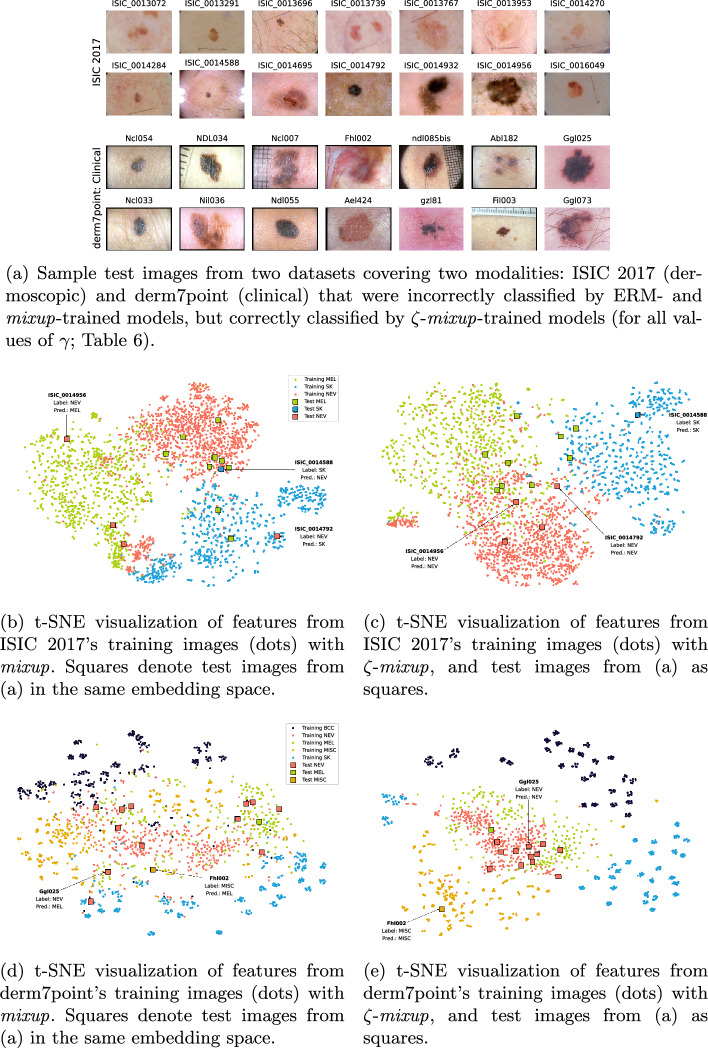
Visualizing how $\zeta$-*mixup* improves performance over *mixup*. Sample images from two skin lesion datasets with different imaging modalities: ISIC 2017 and derm7point. Sample test images from both datasets that were misclassified by *mixup*-augmented models (**a**), when embedded in a 2D space for t-SNE visualization, show that they lie in the vicinity of training samples from classes different from the test images’ labels, leading to wrong predictions (**b**, **d**). On the other hand, with $\zeta$-*mixup*-augmented models, the test images are more likely to be in a region of training samples from the same class as that of the test images (**c**, **e**)

**Fig. 9:**
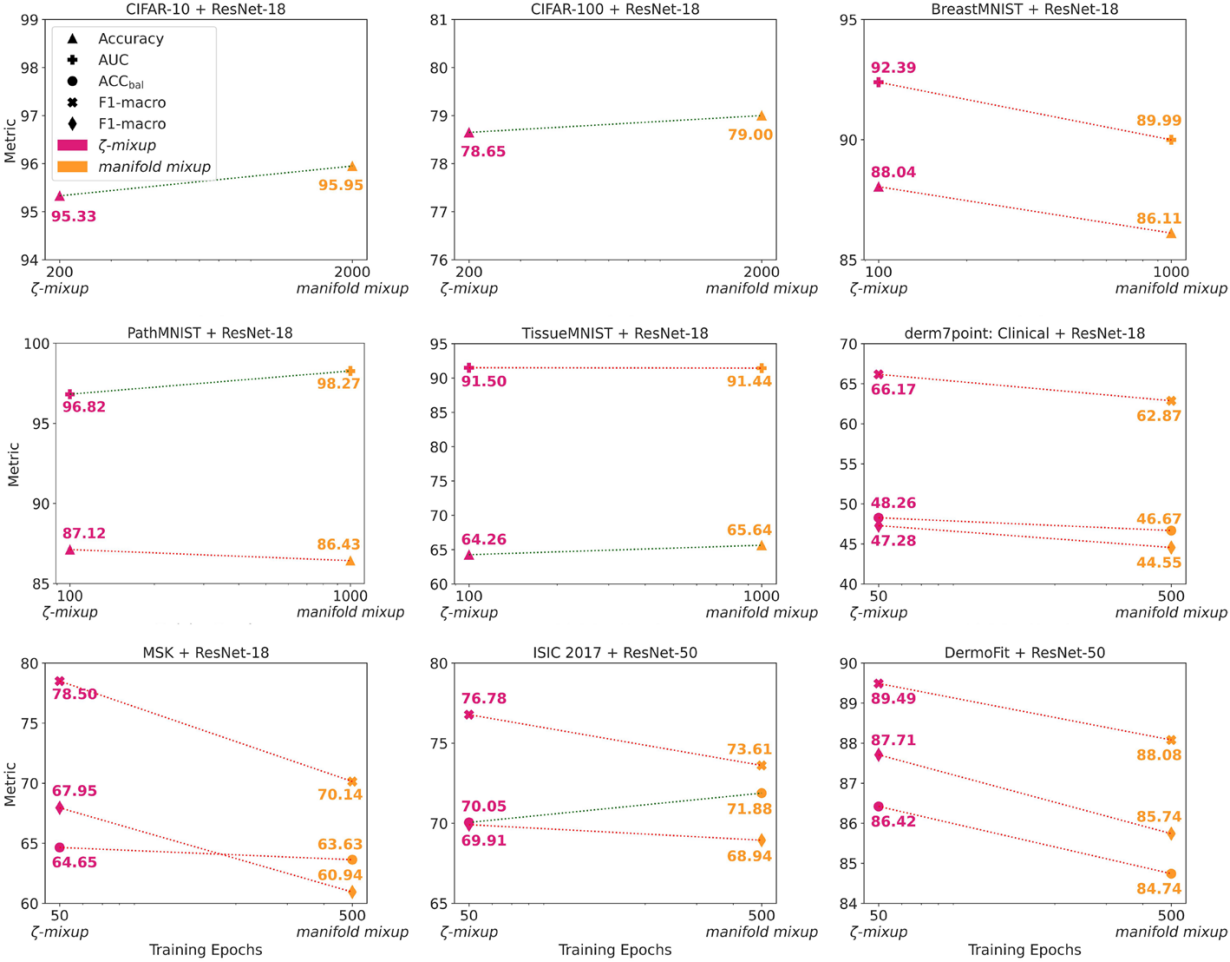
Comparing $\zeta$-*mixup* to *manifold mixup* on nine natural and medical image datasets spanning two model architectures, multiple medical imaging modalities, and image types (RGB and grayscale). All models trained with *manifold mixup* are optimized for $10\times$ the number of epochs compared to their $\zeta$-*mixup* counterparts. We use the same metrics for evaluation as reported in Tables 4, 6 and 7. The dotted lines connecting the pairs of metric values for $\zeta$-*mixup* and *manifold mixup* are color-coded: green indicates that the metric is higher for the model trained with *manifold mixup*, and red denotes vice versa. The metrics reported here are the mean values of three runs for each model. For all metrics, higher values are better. Note that despite being an order of magnitude more computationally expensive, *manifold mixup* does not consistently outperform $\zeta$-*mixup*

**Fig. 10:**
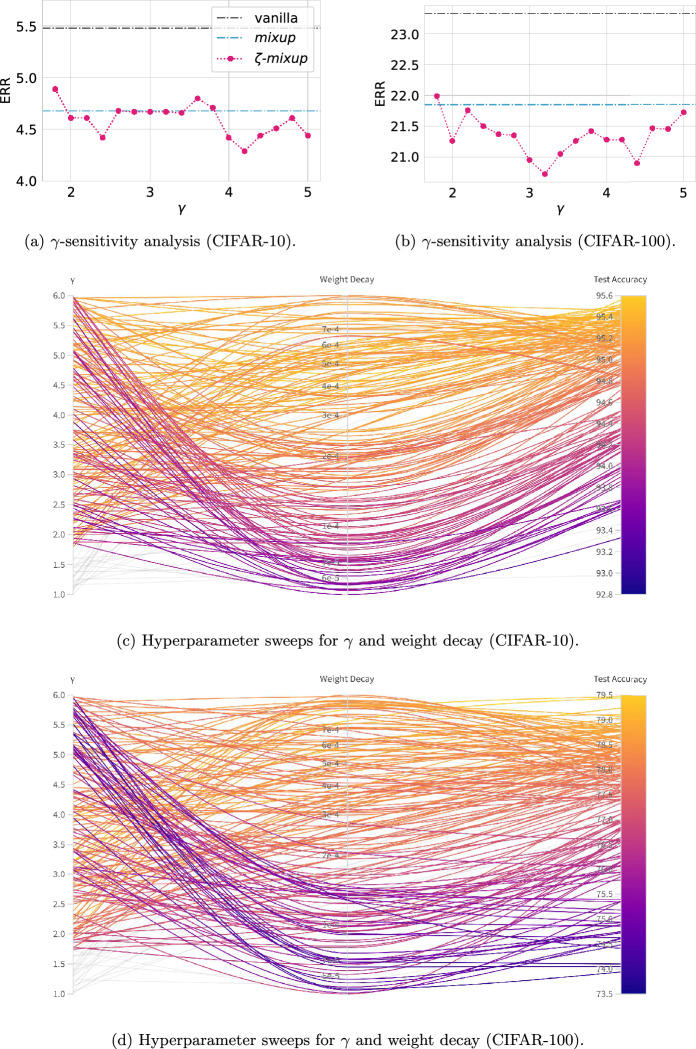
Hyperparameter sensitivity analysis for $\zeta$-*mixup*  on CIFAR-10 and CIFAR-100. In **a**, **b**, $\gamma$ is varied from [1.8, 5.0] and the resulting ERR is shown. In **c**, **d**, 200 models are trained by varying $\gamma$ uniformly in [1.0, 6.0] and weight decay log-uniformly in [5e$-5, 1$e$-3]$. Each model is denoted by a curved line passing through the value of $\gamma$ (left column) and weight decay (middle column) used for training, connecting it to the corresponding model’s test accuracy (right column). The lines are color-coded according to the models’ test accuracy. Models with $\gamma < \gamma _{\textrm{min}}$ are shown in light gray

## Method

### Vicinal risk minimization

Revisiting the concept of risk minimization from Vapnik [50], given ${\mathcal {X}}$ and ${\mathcal {Y}}$ as the input data and the target labels respectively, and a family of functions ${\mathcal {F}}$, the supervised learning setting consists of searching for an optimal function $f \in {\mathcal {F}}: {\mathcal {X}} \rightarrow {\mathcal {Y}}$, which minimizes the expected value of a given loss function ${\mathcal {L}}$ over the data distribution $P(x, y); (x, y) \in ({\mathcal {X}}, {\mathcal {Y}})$. Table 1 lists all the mathematical notations used in this paper. This expected value of the loss, also known as the expected value of the risk, is given by:

1 $$\begin{aligned} R (f) = \int {\mathcal {L}} \left( f(x), y\right) \ P(x, y) \ dx \ dy. \end{aligned}$$

**Table 1:** Summary of notations

Notation	Description	Notation	Description
*x*	Input data sample	${\mathcal {H}}$	Entropy
*y*	Target label sample	${\mathcal {D}}$	Dimensionality of the input space
${\hat{x}}$	Synthesized input data sample	$\textrm{U}$	Uniform distribution
${\hat{y}}$	Synthesized target label sample	$\alpha$	*mixup* hyperparameter
${\mathcal {X}}$	Input data distribution	${\mathcal {M}}$	Data manifold
${\mathcal {Y}}$	Target label distribution	*d*	Intrinsic dimensionality of a manifold
*P*(*x*, *y*)	Data distribution over the input and the target	$N$	Number of samples in a dataset
$P_{\textrm{vic}} (x, y)$	Vicinal data distribution	$T$	Number of samples used for interpolation
${\mathcal {L}}$	Loss function	*m*	Number of samples in a mini-batch
*R*	Risk	$\pi$	${T} \times {T}$ random permutation matrix
$R_{\textrm{emp}}$	Empirical risk	*s*	Randomized ordering of samples
$R_{\textrm{vic}}$	Vicinal risk	$w_i$	Per-sample weight in $\zeta$-*mixup*
$\lambda$	Linear interpolation factor	*C*	Normalization constant for $\zeta$-*mixup* weights
${\mathcal {K}}$	Number of unique classes in the label distribution	$\gamma$	$\zeta$-*mixup* hyperparameter
${\mathcal {S}}$	Label space	$\gamma _{\textrm{min}}$	Minimum value of $\gamma$ to achieve the desirable properties of $\zeta$-*mixup* (see Theorem 1)

**Table 2:** A brief comparison of existing mixing-based data augmentation methods summarizing their key idea, the space in which the interpolation is performed, the number of hyperparameters in the method, and the number of samples used for mixing to generate 1 new sample

Method	Key idea	Interpolation space	Number of hyperparameters	Involves additional optimization	Number of samples mixed
SamplePairing [38]	Linear interpolation of pairs of images with a ratio $\lambda = 0.5$; use labels of the first image	Input	0	✗	2
Between-Class Learning [37]	Linear interpolation of pairs of images from different classes and their labels	Input	0	✗	2
*mixup* [36]	Linear interpolation of pairs of samples and their labels	Input	1 $(\alpha )$	✗	2
CutMix [39]	Paste a rectangular patch from one image onto another; mix labels proportionally	Input	3 $(r_x, r_y, \lambda )$	✗	2
GridMix [40]	Paste a grid-based region from one image onto another; assign a mixed label and grid-based labels	Input	2 (*N*, *p*)	✗	2
*Manifold Mixup* [41]	Linear interpolation of latent representations and their labels	Latent	1 $(\alpha , {\mathcal {S}})$	✗	2
MixFeat [42]	Linear interpolation of latent representations only	Latent	1 $(\sigma )$	✗	2
AdaMixUp [25]	Train an additional network to learn mixing policy from data	Input	0	✓	2
AutoMix [44]	Bi-level optimization for mixed sample generation and mixup classification	Input	3 $(\alpha , l, m)$	✓	2
OptTransMix, AutoMix [43]	Optimization using optimal transport (OptTransMix) in input space or DNNs (AutoMix) in latent space for barycenter learning	Input/Latent	2 $(n, \sigma )$	✓	2
SuperMix [99]	Iterative optimization-based salient masks for mixing	Input	5 $(\alpha , \kappa , k, \sigma , \lambda _s)$	✓	3
Co-Mixup [96]	Iterative optimization-based mixing to maximize data saliency and encourage submodular diversity	Input	6 $(\alpha , \beta , \gamma , \eta , \tau , \omega )$	✓	4
$\zeta$-*mixup* **(Ours)**	*p*-series-weighted convex combination of entire mini-batch of samples and their labels	Input	1 $(\gamma )$	✗	$m (\ge 2)$

For all the methods listed in this table, the variable names of the hyperparameters are listed as they appear in the respective original papers to facilitate easy cross referencing. For *Manifold Mixup*, ${\mathcal {S}}$ denotes the set of eligible layers. Note that some of these methods [25, 43, 44, 96, 99] rely on optimizing additional parameters. Our proposed method, $\zeta$-*mixup*, does not rely on any optimization, and is the only method that mixes up to *m* samples, where *m* is the batch size of the mini-batch

In scenarios when the exact distribution *P*(*x*, *y*) is unknown, such as in practical supervised learning settings with a finite training dataset $\{x_i, y_i\}_{i=1}^{{{N}}}$, the common approach is to minimize the risk w.r.t. the empirical data distribution approximated by using delta functions at each sample,

2 $$\begin{aligned} R_{\textrm{emp}} (f) = \frac{1}{m} \sum _{i=1}^{{{N}}} {\mathcal {L}} \left( f(x_i), y_i\right) , \end{aligned}$$

and this is known as empirical risk minimization (ERM). However, if the data distribution is smooth, as is the case with most real datasets, it is desirable to minimize the risk in the vicinity of the provided samples [50, 51],

3 $$\begin{aligned} R_{\textrm{vic}} (f) = \frac{1}{{\hat{m}}} \sum _{i=1}^{{\hat{m}}} {\mathcal {L}} \left( f(\hat{x_i}), \hat{y_i}\right) , \end{aligned}$$

where $\left\{ ({\hat{x}}, {\hat{y}})\right\} _{i=1}^{{\hat{m}}}$ are points sampled from the vicinity of the original data distribution, also known as the vicinal distribution $P_{\textrm{vic}} (x, y)$. This is known as vicinal risk minimization (VRM) and theoretical analysis [50–52] has shown that VRM generalizes well when at least one of these two criteria are satisfied: (i) the vicinal data distribution $P_{\textrm{vic}} (x, y)$ must be a good approximation of the actual data distribution *P*(*x*, *y*), and (ii) the class ${\mathcal {F}}$ of functions must have a suitably small capacity. Since modern deep neural networks have up to hundreds of millions of parameters, it is imperative that the former criteria is met.

**Table 3:** List of classes from ImageNet and the corresponding WordNet synset IDs in Imagenette and Imagewoof datasets

Imagenette		Imagewoof	
ImageNet class	WordNet synset ID	ImageNet class	WordNet synset ID
tench	n01440764	Australian terrier	n02096294
English springer	n02102040	Border terrier	n02093754
cassette player	n02979186	Samoyed	n02111889
chain saw	n03000684	Beagle	n02088364
church	n03028079	Shih-Tzu	n02086240
French horn	n03394916	English foxhound	n02089973
garbage truck	n03417042	Rhodesian ridgeback	n02087394
gas pump	n03425413	Dingo	n02115641
golf ball	n03445777	Golden retriever	n02099601
parachute	n03888257	Old English sheepdog	n02105641

**Table 4:** Classification error rates (ERR) on NATURAL

Method	CIFAR-10	CIFAR-100	F-MNIST	STL-10	Imagenette	Imagewoof
# images (#classes)	60,000 (10)	60,000 (10)	60,000 (10)	13,000 (10)	13,394 (10)	12,954 (10)
ERM	$5.48 \pm 0.03$	$23.33 \pm 0.09$	$6.11 \pm 0.02$	$25.74 \pm 0.17$	$16.08 \pm 0.15$	$30.92 \pm 0.02$
*mixup*	$4.68 \pm 0.09$	$21.85 \pm 0.07$	$6.04 \pm 0.20$	$25.31 \pm 0.33$	$16.20 \pm 0.03$	$30.80 \pm 0.04$
$\zeta$-*mixup* ($\gamma =2.4$)	$\mathbf{4.42 \pm 0.02}$ ***+*** ***5.56%***	$21.50 \pm 0.04$ ***+*** ***1.60%***	$6.04 \pm 0.04$ *+* *0.00%*	$\mathbf{24.14 \pm 0.10}$ ***+*** ***4.62%***	$\mathbf{15.16 \pm 0.07}$ ***+*** ***6.42%***	$30.72 \pm 0.02$ ***+*** ***0.26%***
$\zeta$-*mixup* ($\gamma =2.8$)	${{\underline{4.67 \pm 0.05}}}$ ***+*** ***0.21%***	${{\underline{21.35 \pm 0.02}}}$ ***+*** ***2.29%***	$\mathbf{5.70 \pm 0.07}$ ***+*** ***5.63%***	${{\underline{24.82 \pm 0.03}}}$ ***+*** ***1.94%***	${{\underline{15.62 \pm 0.07}}}$ ***+*** ***3.58%***	$\mathbf{30.21 \pm 0.05}$ ***+*** ***1.92%***
$\zeta$-*mixup* ($\gamma =4.0$)	$\mathbf{4.42 \pm 0.01}$ ***+*** ***5.56%***	$\mathbf{21.28 \pm 0.02}$ ***+*** ***2.61%***	${{\underline{5.89 \pm 0.04}}}$ ***+*** ***2.48%***	$24.92 \pm 0.22$ ***+*** ***1.54%***	$15.92 \pm 0.07$ ***+*** ***1.73%***	${{\underline{30.67 \pm 0.03}}}$ ***+*** ***0.42%***

The lowest and the second lowest errors are formatted with bold and underline respectively. Percentage relative improvements over *mixup*  are shown in green. ERRs are reported as mean ± standard deviation over 3 runs. Lower values are better

**Table 5:** Classification error rate (ERR) improvements on CIFAR-10 and CIFAR-100 datasets with $\zeta$-*mixup*  applied in conjunction with CutMix

Method	ResNet-18	ResNet-50	MobileNetV2	EfficientNet-B0
CIFAR-10				
CutMix	$4.13 \pm 0.01$	$4.08 \pm 0.12$	$8.97 \pm 0.08$	${9.99 \pm 0.29}$
+ $\zeta$-*mixup*	$\varvec{3.84 \pm 0.08}$ ***+*** ***7.02%***	$\varvec{3.61 \pm 0.06}$ ***+*** ***11.52%***	$\varvec{8.18 \pm 0.09}$ ***+*** ***8.81%***	$\varvec{9.15 \pm 0.08}$ ***+*** ***8.41%***
CIFAR-100				
CutMix	${19.97 \pm 0.07}$	$18.99 \pm 0.08$	$28.93 \pm 0.18$	$31.55 \pm 0.15$
+ $\zeta$-*mixup*	$\varvec{19.54 \pm 0.06}$ ***+*** ***2.15%***	$\varvec{18.86 \pm 0.04}$ ***+*** ***0.68%***	$\varvec{28.31 \pm 0.25}$ ***+*** ***2.14%***	$\varvec{30.73 \pm 0.07}$ ***+*** ***2.29%***

The lowest errors are formatted with bold. Percentage relative improvements over using only CutMix are shown in green. ERRs are reported as mean ± standard deviation over 3 runs. Lower values are better

### Data augmentation

A popular example of VRM is the use of data augmentation for training deep neural networks. For example, applying geometric and intensity-based transformations to images leads to a diverse training dataset allowing the prediction models to generalize well to unseen samples [35]. However, the assumption of these transformations that points sampled in the vicinity of the original data distribution share the same class label is rather limiting and does not account for complex interactions (e.g.,  proximity relationships) between class-specific data distributions in the input space. Recent approaches based on convex combinations of pairs of samples to synthesize new training samples aim to alleviate this by allowing the model to learn smoother decision boundaries [41]. Consider the general ${\mathcal {K}}$-class classification task. *mixup* [36] synthesizes a new training sample $({\hat{x}}, {\hat{y}})$ from training data samples $(x_i, y_i)$ and $(x_j, y_j)$ as

4 $$\begin{aligned} \begin{aligned} {\hat{x}} = \lambda x_i + (1 - \lambda ) x_j \\ {\hat{y}} = \lambda y_i + (1 - \lambda ) y_j. \end{aligned} \end{aligned}$$

**Table 6:** Classification performance evaluated on SKIN

Dataset	ISIC 2016^†^						ISIC 2017^†^					
#images (#classes)	1279 (2)						2750 (3)					
Method	ResNet-18			ResNet-50			ResNet-18			ResNet-50		
	ACC_bal_	F1-micro	F1-macro	ACC_bal_	F1-micro	F1-macro	ACC_bal_	F1-micro	F1-macro	ACC_bal_	F1-micro	F1-macro
ERM	70.44%	0.7836	0.6865	71.75%	0.8127	0.7121	69.31%	0.7383	0.6720	68.20%	0.6867	0.6361
*mixup*	71.77%	0.7968	0.7017	72.08%	0.8179	0.7175	71.60%	0.7333	0.6756	71.51%	0.7433	0.6979
$\zeta$-*mixup* (2.4)	**74.53%**	0.8417	0.7180	71.52%	**0.8654**	0.7492	**73.02%**	0.7483	0.6965	**72.91%**	**0.7783**	**0.7099**
$\zeta$-*mixup* (2.8)	73.03%	**0.8654**	**0.7588**	**72.20%**	0.8602	**0.7493**	72.33%	**0.7633**	**0.7068**	69.99%	0.7733	0.7028
$\zeta$-*mixup* (4.0)	72.27%	0.7968	0.7043	72.11%	0.8391	0.7151	70.93%	0.7567	0.6815	72.39%	0.7517	0.6963

Dataset	ISIC 2018^†^						MSK^†^					
#images (#classes)	10,015 (5)						3551 (4)					
Method	ResNet-18			ResNet-50			ResNet-18			ResNet-50		
	ACC_bal_	F1-micro	F1-macro	ACC_bal_	F1-micro	F1-macro	ACC_bal_	F1-micro	F1-macro	ACC_bal_	F1-micro	F1-macro
ERM	84.31%	0.8756	0.8122	81.28%	0.8653	0.7982	62.35%	0.6986	0.5999	63.86%	0.7873	0.6586
*mixup*	83.96%	0.8394	0.7767	85.65%	0.8601	0.8064	63.59%	0.7423	0.6404	65.62%	0.7958	0.6434
$\zeta$-*mixup* (2.4)	**87.20%**	**0.8964**	**0.8441**	84.75%	0.8653	0.8112	**65.52%**	0.7746	0.6475	65.23%	**0.8056**	**0.6875**
$\zeta$-*mixup* (2.8)	84.67%	0.8756	0.8066	86.59%	0.9016	0.8333	64.87%	**0.7845**	**0.6883**	**65.94%**	0.7930	0.6704
$\zeta$-*mixup* (4.0)	83.63%	0.8808	0.8062	**89.18%**	**0.9223**	**0.8718**	62.39%	0.6930	0.6006	65.33%	0.7817	0.6587

Dataset	UDA^†^						DermoFit^‡^					
#images (#classes)	601 (2)						1,300 (5)					
Method	ResNet-18			ResNet-50			ResNet-18			ResNet-50		
	ACC_bal_	F1-micro	F1-macro	ACC_bal_	F1-micro	F1-macro	ACC_bal_	F1-micro	F1-macro	ACC_bal_	F1-micro	F1-macro
ERM	67.46%	0.7000	0.6666	66.85%	0.6917	0.6593	80.43%	0.8269	0.8120	83.24%	0.8500	0.8316
*mixup*	69.38%	0.7167	0.6851	67.27%	0.7167	0.6727	81.17%	0.8577	0.8302	84.37%	0.8500	0.8406
$\zeta$-*mixup* (2.4)	**70.54%**	**0.8000**	**0.7272**	68.39%	0.7417	0.6900	82.57%	0.8692	0.8419	86.26%	0.8615	0.8491
$\zeta$-*mixup* (2.8)	70.22%	0.7667	0.7127	**70.92%**	**0.7667**	**0.7176**	83.50%	0.8731	0.8459	85.91%	0.8962	0.8765
$\zeta$-*mixup* (4.0)	67.88%	0.7250	0.6800	67.59%	0.7500	0.6865	**83.94%**	**0.8769**	**0.8514**	**88.16%**	**0.9115**	**0.9008**

Dataset	derm7point: Clinical^‡^						derm7point: Dermoscopic^†^					
#images (#classes)	1,011 (5)						1,011 (5)					
Method	ResNet-18			ResNet-50			ResNet-18			ResNet-50		
	ACC_bal_	F1-micro	F1-macro	ACC_bal_	F1-micro	F1-macro	ACC_bal_	F1-micro	F1-macro	ACC_bal_	F1-micro	F1-macro
ERM	42.08%	0.5297	0.3797	42.15%	0.6485	0.4328	54.79%	0.7030	0.5670	55.46%	0.7574	0.5819
*mixup*	46.68%	0.5941	0.4392	45.57%	0.6485	0.4474	55.38%	0.7376	0.5683	62.08%	**0.7772**	0.6419
$\zeta$-*mixup* (2.4)	47.82%	0.6782	0.4833	46.63%	0.6436	0.4239	55.88%	0.7525	**0.5914**	**64.59%**	0.7376	0.6406
$\zeta$-*mixup* (2.8)	**48.91%**	0.6089	0.4496	**48.36%**	0.6733	**0.5122**	**56.41%**	**0.7574**	0.5700	62.98%	0.7624	0.6552
$\zeta$-*mixup* (4.0)	46.93%	**0.7030**	**0.4902**	45.95%	**0.6881**	0.4828	55.45%	0.7178	0.5618	62.58%	**0.7772**	**0.6622**

Dataset	PH2^†^						MED-NODE^‡^					
#images (#classes)	200 (2)						170 (2)					
Method	ResNet-18			ResNet-50			ResNet-18			ResNet-50		
	ACC_bal_	F1-micro	F1-macro	ACC_bal_	F1-micro	F1-macro	ACC_bal_	F1-micro	F1-macro	ACC_bal_	F1-micro	F1-macro
ERM	84.38%	0.8000	0.8438	84.38%	0.9000	0.8438	75.00%	0.7941	0.7589	74.64%	0.7647	0.7509
*mixup*	85.94%	0.9250	0.8769	85.94%	0.8500	0.8000	80.36%	0.7941	0.7925	**81.79%**	**0.8235**	**0.8179**
$\zeta$-*mixup* (2.4)	85.94%	0.9250	0.8769	**87.50%**	**0.9500**	**0.9134**	79.29%	0.7941	0.7986	80.71%	**0.8235**	0.8132
$\zeta$-*mixup* (2.8)	**96.88%**	**0.9500**	**0.9283**	**87.50%**	**0.9500**	**0.9134**	**82.86%**	**0.8235**	**0.8211**	**81.79%**	**0.8235**	**0.8179**
$\zeta$-*mixup* (4.0)	85.94%	0.9250	0.8769	**87.50%**	**0.9500**	**0.9134**	81.79%	**0.8235**	0.8179	80.71%	**0.8235**	0.8132

^†^ and ^‡^ denote dermoscopic and clinical skin lesion images respectively. The evaluation metrics are balanced accuracy (‘ACC_bal_’), micro-averaged F1 score (‘F1-micro’), and macro-averaged F1 score (‘F1-macro’). Higher values are better for all the metrics. The highest and the second highest values of each metric have been formatted with bold and underline respectively

**Table 7:** Classification performance (AUC and ACC) evaluated on MEDMNIST

Dataset	PathMNIST		DermaMNIST		OCTMNIST		PneumoniaMNIST		BloodMNIST	
#images (#classes)	107,180 (9)		10,015 (7)		109,309 (4)		5,856 (2)		17,092 (8)	
Method	AUC	ACC	AUC	ACC	AUC	ACC	AUC	ACC	AUC	ACC
ERM	0.962	84.4%	0.899	72.1%	**0.951**	70.8%	0.947	80.3%	0.995	92.9%
*mixup*	0.959	77.5%	0.897	72.2%	0.945	70.5%	0.945	75.4%	0.994	94.4%
$\zeta$-*mixup* ($\gamma =2.8$)	**0.969**	**87.6%**	**0.911**	**73.3%**	0.918	**72.8%**	**0.951**	**80.9%**	**0.997**	**95.2%**

Dataset	TissueMNIST		BreastMNIST		OrganMNIST_A		OrganMNIST_C		OrganMNIST_S	
#images (#classes)	236,386 (8)		780 (2)		58,850 (11)		23,660 (11)		25,221 (11)	
Method	AUC	ACC	AUC	ACC	AUC	ACC	AUC	ACC	AUC	ACC
ERM	0.911	62.7%	0.897	85.9%	0.995	92.1%	0.990	88.9%	0.967	76.2%
*mixup*	0.911	63.2%	0.914	76.2%	0.995	**93.1%**	0.990	89.9%	0.966	72.7%
$\zeta$-*mixup* ($\gamma =2.8$)	**0.918**	**63.9%**	**0.928**	**87.2%**	**0.996**	92.7%	**0.991**	**91.0%**	**0.969**	**77.1%**

The evaluation metrics are the area under the ROC curve (‘AUC’) and the classification accuracy (‘ACC’). Higher values are better for both the metrics. The highest values of each metric have been formatted with bold

**Table 8:** Ablation study to analyze the contribution of both the components of $\zeta$-*mixup* when used in isolation on CIFAR-100: mixing more than 2 samples and using weights from a normalized *p*-series for the mixing

Mixes $>2$ samples	Uses normalized *p*-series weights for mixing	Method name	CIFAR-100 ERR
✗ $T=2$	✗ weights from a Beta($\alpha$, $\alpha$) distribution; $\alpha = 1$	*mixup*	$21.85 \pm 0.07$
✗ $T=2$	✓ weights from a normalized *p*-series	-	$21.77 \pm 0.17$
✓ $T=m$	✗ weights from a Dirichlet($\alpha$) distribution; $\alpha = 1$	-	$94.69 \pm 0.08$
✓ $T=m$	✓ weights from a normalized *p*-series	$\zeta$-*mixup*	$\varvec{21.35 \pm 0.02}$

Note that since $\zeta$-*mixup* is a generalization of *mixup* (Theorem 2), $\zeta$-*mixup* without both these components reduces to *mixup* (first row). Next, modifying *mixup* to use $\zeta$-*mixup* ’s weighting scheme but only for 2 samples (second row) outperforms *mixup*, but is inferior to $\zeta$-*mixup*. On the other hand, mixing the entire batch ($T=m$) but with a Dirichlet distribution leads to extremely poor performance (third row). Finally, using both of these components, i.e., $\zeta$-*mixup*, leads to the best performance

**Table 9:** Benchmarking various methods for training models on CIFAR-100, STL-10, Imagenette, and for augmenting a batch of 32 RGB images of $224 \times 224$ spatial resolution

Method		CIFAR-100 (200 epochs)	STL-10 (200 epochs)	Imagenette (80 epochs)	[32, 3, 224, 224] torch.Tensor
Wall Time	*mixup* [36]	1h 20m ± 23s	24m 59s ± 16.9s	45m 39s ± 8.5s	745$\mu$s ± 9.55$\mu$s
	$\zeta$-*mixup*	1h 20m ± 17s	24m 58s ± 4.6s	45m 34s ± 14.1s	345$\mu$s ± 2.53$\mu$s
	CutMix [39]	1h 22m ± 13s	†	†	176$\mu$s ± 1.4$\mu$s
	CutMix [39] + $\zeta$-*mixup*	1h 22m ± 9s	†	†	169$\mu$s ± 757ns
	*Manifold Mixup* [41]	16h 15m (2000 epochs)	‡	‡	‡‡
	Co-Mixup [96]	16h 35m (300 epochs)	‡	‡	‡‡
	Local synthetic instances [57]	§	§	§	38.7ms ± 1.33 ms

Note that *mixup*, $\zeta$-*mixup*, CutMix, and CutMix + $\zeta$-*mixup* require 200 epochs of training for CIFAR-100, whereas *Manifold Mixup* and Co-Mixup require 2000 and 300 epochs respectively. CutMix experiments were performed on CIFAR-10 and CIFAR-100, and training times on STL-10 and Imagenette were not available from the original paper either (†). Similarly, given the large computational cost for *Manifold Mixup* and Co-Mixup, we did not train them on STL-10 and Imagenette, and their training times are missing from the respective paper too (‡). We also were unable to benchmark these two methods on a batch of 32 images (last column; ‡‡) since these methods require a DNN forward pass and gradients respectively for augmentation. Finally, the local synthetic instances method [57] is not optimized for training DNNs (§), as it is two orders of magnitude slower than $\zeta$-*mixup* (see last column)

where $\lambda \in [0, 1]$. The labels $y_i$, $y_j$ are converted to one-hot encoded vectors to allow for linear interpolation between pairs of labels. However, as we show in our experiments ("Results and Discussion" Section), *mixup*  leads to the synthesized points being sampled off the data manifold (Fig. 1 (a)).

### $\zeta$-*mixup*  formulation

Going back to the ${\mathcal {K}}$-class classification task, suppose we are given a set of ${T}$ points $\{x_i\}_{i=1}^{{T}}$ in a ${\mathcal {D}}$-dimensional ambient space $\mathbb {R^{{\mathcal {D}}}}$ with the corresponding labels $\{y_i\}_{i=1}^{{{T}}}$ in a label space ${{\mathcal {S}}} = \{l_1, \cdots , l_{\mathcal {K}}\} \in \mathbb {R^{{\mathcal {K}}}}$. Keeping in line with the manifold hypothesis [53, 54], which states that complex data manifolds in high-dimensional ambient spaces are actually made up of samples from manifolds with low intrinsic dimensionalities, we assume that the ${T}$ points are samples from ${\mathcal {K}}$ manifolds $\{{\mathcal {M}}_i\}_{i=1}^{{\mathcal {K}}}$ of intrinsic dimensionalities $\{d_i\}_{i=1}^{{\mathcal {K}}}$, where $d_i<< D \ \forall i \in [1, {\mathcal {K}}]$ (Fig. 1a). We seek an augmentation method that facilitates a denser sampling of each intrinsic manifold ${\mathcal {M}}_i$, thus generating more real and more diverse samples with richer labels. Following Wood et al. [55, 56], we consider criteria 1 through 3 below for evaluating the quality of synthetic data:

1. realism: allowing the generation of correctly labeled synthetic samples close to the original samples, ensuring the realism of the synthetic samples,
2. diversity: facilitating the generation of more diverse synthetic samples by allowing exploration of the input space, and
3. label richness when generating synthetic samples while still staying on the manifold of realistic samples.

In addition to the above three criteria, we also aim for the following two objectives:

4. valid probabilistic labels from combinations of samples along with
5. computationally efficient (e.g.,  avoiding inter-sample distance calculations) augmentation of training batches.

To this end, we propose to synthesize a new sample ${({\hat{x}}_k, {\hat{y}}_k)}$ as

5 $$\begin{aligned} \begin{aligned} {{\hat{x}}_k} = \sum _{i=1}^{{T}} w_i x_i \\ {{\hat{y}}_k} = \sum _{i=1}^{{T}} w_i y_i, \end{aligned} \end{aligned}$$

where $w_i$s are the weights assigned to the ${T}$ samples. One such weighting scheme that satisfies the aforementioned requirements consists of sample weights from the terms of a *p*-series, i.e.,  $w_i = i^{-p}$, which is a convergent series for $p \ge 1$. Since this implies that the weight assigned to the first sample will be the largest, we want to randomize the order of the samples to ensure that the synthetic samples are not all generated near one original sample. Therefore, building upon the idea of local synthetic instances initially proposed for the augmentation of connectome dataset [57], we adopt the following formulation: given ${T}$ samples (where $2 \le {T} \le m \le {N}$ and thus, theoretically, the entire dataset), an ${T} \times {T}$ random permutation matrix $\pi$, and the resulting randomized ordering of samples $s = \pi [1, 2, \dots , {T}]^\textsf{T}$, the weights are defined as

6 $$\begin{aligned} w_i = \frac{s_i^{-\gamma }}{C}, \ \ i \in [1, {T}], \end{aligned}$$

where *C* is the normalization constant and $\gamma$ is a hyperparameter. As we show in our experiments later, $\gamma$ allows us to control how far the synthetic samples can stray away from the original samples. Moreover, in order to ensure that $y_k$ in Eq. (5) is a valid probabilistic label, $w_i$ must satisfy $w_i \ge 0 \ \forall i$ and $\sum _{i=1}^{{T}} w_i = 1$. Accordingly, we use $L_1$-normalization and $C = \sum _{j=1}^{{T}} j^{-\gamma }$ is the ${T}$-truncated Riemann zeta function [58] $\zeta (z)$ evaluated at $z=\gamma$, and call our method $\zeta$-*mixup*. The algorithmic formulation of $\zeta$-*mixup* is presented in Algorithm 1.

An illustration of $\zeta$-*mixup*  for ${T}=3, {\mathcal {D}}=3, d_1 = d_2 = d_3 =2$ is shown in Fig. 1a. Notice how despite generating convex combinations of samples from disjoint manifolds, the resulting synthetic samples are close to the original ones. A similar observation can be made for ${T}=4$ and ${T}=8$ is shown in Fig. 1c. Figure 1d shows an overview of how $\zeta$-*mixup* generates new samples for a mini-batch of size $m = {T} = 4$, with 3 classes (${\mathcal {K}} = 3$) and the hyperparameter $\gamma = 2.4$.

Since there exist ${T}!$ possible ${T} \times {T}$ random permutation matrices, given ${T}$ original samples, $\zeta$-*mixup*  can synthesize ${T}!$ new samples for a single value of $\gamma$, as compared to *mixup*  which can only synthesize 1 new sample per sample pair for a single value of $\lambda$.

As a result of the aforementioned formulation, $\zeta$-*mixup*  presents two desirable properties that we present in the following 2 theorems. Theorem 1 states that for all values of $\gamma \ge \gamma _{\textrm{min}}$, the weight assigned to one sample is greater than the sum of the weights assigned to all the other samples in a batch, thus implicitly introducing the desired notion of linearity in only the locality of the original samples. Theorem 2 states the equivalence of *mixup*  and $\zeta$-*mixup*  and establishes the former as a special case of the latter.

#### Theorem 1

*For* $\gamma \ge \gamma _{\textrm{min}}= 1.72865$*, the weight assigned to one sample dominates all other weights, i.e.,* $\forall \ \gamma \ge 1.72865$,

7 $$\begin{aligned} w_1 > \sum _{i=2}^{{T}} w_i. \end{aligned}$$

#### Proof

Let us consider the case when ${T} \rightarrow \infty$. We need to find the value of $\gamma$ such that

8 $$\begin{aligned}&w_1 > \sum _{i=2}^\infty w_i \end{aligned}$$

9 $$\begin{aligned} \Rightarrow&\ \frac{1^{-\gamma }}{C} > \sum _{i=2}^\infty \frac{i^{-\gamma }}{C} ; \ \ C = \sum _{j=1}^\infty j^{-\gamma }, \end{aligned}$$

10 $$\begin{aligned} \Rightarrow&\ 1^{-\gamma }> \sum _{i=2}^\infty i^{-\gamma } \ (\textrm{since } \ C > 0), \end{aligned}$$

11 $$\begin{aligned} \Rightarrow&\ 1^{-\gamma } + 1^{-\gamma } > 1^{-\gamma } + \sum _{i=2}^\infty i^{-\gamma }, \end{aligned}$$

12 $$\begin{aligned} \Rightarrow&\ 2 > \sum _{i=1}^\infty i^{-\gamma }. \end{aligned}$$

Note that $\sum _{i=1}^\infty i^{-\gamma } = \zeta (\gamma )$ is the Riemann zeta function at $\gamma$. Using a solver, we get $\gamma \ge 1.72865$. Therefore, $\forall \ \gamma \ge \gamma _{\textrm{min}}= 1.72865$,

13 $$\begin{aligned} w_1> \sum _{i=2}^\infty w_i> \sum _{i=2}^{{T}} w_i \Rightarrow w_1 > \sum _{i=2}^{{T}} w_i. \end{aligned}$$

$$\square $$

#### Theorem 2

*For* ${T} = 2$* and* $\gamma = \log _2 \left( \frac{\lambda }{1-\lambda }\right)$, $\zeta$-*mixup*  *simplifies to mixup*.

#### Proof

When ${T}=2$, $\zeta$-*mixup*  (Eq. 5) generates new samples by

14 $$\begin{aligned} \begin{aligned} x_k = \sum _{i=1}^2 w_i x_i = w_1 x_1 + w_2 x_2 \\ y_k = \sum _{i=1}^2 w_i y_i = w_1 y_1 + w_2 y_2, \end{aligned} \end{aligned}$$

where

15 $$\begin{aligned} w_1 = \frac{1^{-\gamma }}{1^{-\gamma } + 2^{-\gamma }}; \ \ w_2 = \frac{2^{-\gamma }}{1^{-\gamma } + 2^{-\gamma }}. \end{aligned}$$

For this to be equivalent to *mixup*  (Eq. 4), we should have

16 $$\begin{aligned} w_1 = \lambda ; \ \ w_2 = 1 - \lambda . \end{aligned}$$

Solving for $\gamma$, we have

17 $$\begin{aligned} w_1&= \frac{1^{-\gamma }}{1^{-\gamma } + 2^{-\gamma }} = \lambda \end{aligned}$$

18 $$\begin{aligned}&\Rightarrow \frac{1}{1 + 2^{-\gamma }} = \lambda \end{aligned}$$

19 $$\begin{aligned}&\Rightarrow 2^{-\gamma } = \frac{1-\lambda }{\lambda } \end{aligned}$$

20 $$\begin{aligned} \Rightarrow \gamma = - \log _2 \left({ \frac{1-\lambda }{\lambda }}\right) = \log _2 \left( {\frac{\lambda }{1-\lambda }}\right) . \end{aligned}$$

$$\square $$

## Datasets and experimental details

### Synthetic data

To emulate realistic settings where class distributions are not always necessarily linearly separable, we first generate two-class distributions of $2^9 = 512$ samples with non-linear class boundaries in the shape of interleaving crescents (CRESCENTS) and spirals (SPIRALS), and add Gaussian noise with zero mean and standard deviation $\sigma = 0.1$ to the points as shown in the “Input” column of Fig. 2a. Next, moving on to higher dimensional spaces, we generate synthetic data distributed along a helix. In particular, we sample $2^{13}$ = 8,192 points off a 1-D helix embedded in $\mathbb {R}^3$ (see the “Input” column of Fig. 2b) and, as a manifestation of low-D manifolds lying in high-D ambient spaces, a 1-D helix in $\mathbb {R}^{12}$. This is done in accordance with the manifold hypothesis [53, 54] which states that complex data manifolds in high-dimensional ambient spaces (e.g.,  3 dimensions in Fig. 2b) are actually made up of samples from a manifold with a low intrinsic dimensionality (i.e.,  1-dimensional helix in Fig. 2b).

### Natural image datasets (NATURAL)

Broadly speaking, natural images are those acquired by standard RGB cameras in a “reasonably ordinary environment” [59] whereas medical images are acquired with specialized imaging equipment. We use this distinction between natural images and medical images to highlight the differences in what these two broad categories of images encode [60–62]. In this paper, we use MNIST [26], CIFAR-10 and CIFAR-100 [63], Fashion-MNIST (F-MNIST) [64], STL-10 [65], and, to evaluate models on real-world images but with faster training times, two 10-class subsets of the standard ImageNet [5]: Imagenette and Imagewoof [66].

F-MNIST, just like MNIST, has $28 \times 28$ grayscale images. Unlike the CIFAR datasets which have RGB images with $32 \times 32$ spatial resolution, STL-10 consists of RGB images with a higher $96 \times 96$ resolution and also has fewer training images than testing images per class. Finally, Imagenette and Imagewoof are 10-class subsets of the standard ImageNet [5] dataset allowing for evaluating models on natural image datasets but with more realistic training times and computational costs. The list of ImageNet classes and the corresponding synset IDs from WordNet in both these datasets are shown in Table 3. Both the datasets have standardized training and validation partitions.

#### Training details

Because of the ease with which modern deep neural networks can achieve very high classification accuracy on the MNIST dataset, we reserve its usage to visualization purposes only and use the other 6 datasets for training and evaluating deep classification models. For all the datasets, we train and validate deep models with the ResNet-18 architecture [16] on the standard training and validation partitions and use random horizontal flipping for data augmentation. We report the overall accuracy as the metric since the datasets have balanced class distributions.

For CIFAR-10, CIFAR-100, F-MNIST, and STL-10, the ResNet-18 models are trained on the original image resolutions, whereas for Imagenette and Imagewoof, the images are resized to $224 \times 224$. For CIFAR-10, CIFAR-100, F-MNIST, the models are trained for 200 epochs with an initial learning rate of 0.1, which is decayed by a multiplicative factor of 0.2 at $80{\textrm{th}}$, $120{\textrm{th}}$, and $160{\textrm{th}}$ epochs, with batches of 128 images for CIFAR datasets and 32 images for F-MNIST. For STL-10, the models are trained for 120 epochs with a batch size of 32 and an initial learning rate of 0.1, which is decayed by a multiplicative factor of 0.2 at $80{\textrm{th}}$ epoch. Finally, for Imagenette and Imagewoof, the models are trained for 80 epochs with a batch size of 32 and an initial learning rate of 0.01, which is decayed by a multiplicative factor of 0.2 at $25{\textrm{th}}$, $50{\textrm{th}}$, and $65{\textrm{th}}$ epochs. All models are optimized using cross entropy loss and mini-batch stochastic gradient descent (SGD) with Nesterov momentum of 0.9 and a weight decay of 5e$-4$.

Since $\zeta$-*mixup* can interpolate between samples at both image- and patch-levels, we carry out an additional set of experiments to evaluate $\zeta$-*mixup* ’s performance when used in conjunction with other orthogonal augmentation techniques. In particular, we assess if using $\zeta$-*mixup* along with CutMix outperforms using only CutMix. We perform these experiments on the CIFAR-10 and CIFAR-100 datasets and with 4 model architectures: ResNet-18 [16], ResNet-50 [16], MobileNetV2 [67], and EfficientNet-B0 [68]. All the models are trained for 200 epochs with an initial learning rate of 0.1, which is decayed by a multiplicative factor of 0.2 at $100{\textrm{th}}$ and $150{\textrm{th}}$ epochs, and with batches of 128 images. As before, we use the cross entropy loss and SGD with Nesterov momentum of 0.9 and a weight decay of 5e$-4$ to optimize the classification models.

### Skin lesion diagnosis datasets (SKIN)

Next, we move to the medical image diagnosis task and focus on skin lesion classification. Skin lesion imaging has 2 pre-dominant modalities: clinical images and dermoscopic images. While both capture RGB images, clinical images consist of close-up lesion images acquired with consumer-grade cameras, whereas dermoscopic images are acquired using a dermatoscope which allows for identification of detailed morphological structures [69] along with fewer imaging-related artifacts [70]. We use 10 skin lesion image diagnosis datasets: International Skin Imaging Collaboration (ISIC) 2016 [71], ISIC 2017 [72], ISIC 2018 [73, 74], Memorial Sloan-Kettering Cancer Center datasets (MSK-1 through MSK-5, collectively known as MSK) [75], UDA [75], DermoFit ^†^ [76], derm7point-$\{C^{\dagger }, D\}$ [77], PH2 [78], and MED-NODE^†^ [79]. The derm7point dataset [77] contains multi-modal images and are therefore 2 datasets: derm7point-C^†^ (containing clinical images) and derm7point-D (containing dermoscopic images). All the datasets have dermoscopic images, except those denoted by a $\dagger$.

#### Training details

For all the datasets, we train classification models with the ResNet-18 and the ResNet-50 [16] architectures. For data augmentation, we take a square center-crop of the image with edge length equal to 0.8* *min*(height, width) and then resize it to $256 \times 256$ spatial resolution. The ISIC 2016, 2017, and 2018 come with standardized partitions that we use for training and evaluating our models, and for the other 7 datasets, we perform a stratified split in the ratio of training : validation : testing $::$ 70 : 10 : 20. Given the inherent class imbalance in these datasets, we report three evaluation metrics which take class imbalance into account: balanced accuracy (i.e.,  macro-averaged recall per class) [80] and micro- and macro-averaged F1 scores.

For all the datasets, we use the 5-class diagnosis labels used in the original dataset paper and in the literature [77, 81, 82]: “basal cell carcinoma”, “nevus”, “melanoma”, “seborrheic keratosis”, and “others”.

For all the datasets except ISIC 2018, we use a batch size of 32 images and train the models for 50 epochs with an initial learning rate of 0.01, which was decayed by a multiplicative factor of 0.1 every 10 epochs. Given that the ISIC 2018 dataset is considerably larger, we train it for 20 epochs with 32 images in a batch and an initial learning rate of 0.01, which was decayed by a multiplicative factor of 0.1 every 4 epochs. As with experiments with the natural image datasets, all models are optimized using cross entropy loss and SGD with Nesterov momentum of 0.9 and a weight decay of 5e$-4$.

### Datasets of other medical imaging modalities (MEDMNIST)

To evaluate our models on multiple medical imaging modalities, we use 10 datasets from the MedMNIST Classification Decathlon [83]: PathMNIST^‡^ (histopathology images [84]), DermaMNIST^‡^ (multi-source images of pigmented skin lesions [74]), OCTMNIST (optical coherence tomography (CT) images [85]), PneumoniaMNIST (pediatric chest X-ray images [85]), BloodMNIST^‡^ (microscopic peripheral blood cell images [86]), TissueMNIST (microscopic images of human kidney cortex cells [87]), BreastMNIST (breast ultrasound images), and OrganMNIST_{A, C, S} (axial, coronal, and sagittal views respectively of 3D CT scans [88, 89]). Datasets denoted by $\ddagger$ consist of RGB images, others consist of grayscale images.

#### Training details

For all the datasets, we train and evaluate classification models with the ResNet-18 architecture on the standard training, validation, and testing partitions. The images are used in their original $28 \times 28$ spatial resolution, and the evaluation metrics reported are the same as in the original dataset paper [83]: overall accuracy and area under the ROC curve.

For all the datasets, we use a learning rate of 0.01 and following the original paper [83], we use cross entropy loss with SGD on batches of 128 images to optimize the classification models.

## Results and discussion

We present experimental evaluation on controlled synthetic (1-D manifolds in 2-D and 3-D, 3-D manifolds in 12-D) and on 26 real-world natural and medical image datasets of various modalities. We evaluate the quality of $\zeta$-*mixup* ’s outputs: directly, by assessing the realism, label correctness, diversity, richness [55, 56], and preservation of intrinsic dimensionality of the generated samples; as well as indirectly, by assessing the effect of the samples on the performance of downstream classification tasks. For classification tasks, we compare models trained with $\zeta$-*mixup* ’s outputs against those trained with traditional data augmentation techniques (ERM) and with *mixup* ’s outputs.

Since $\zeta$-*mixup*  and *mixup*  are used to perform data augmentation on-the-fly while training DNNs, it is imperative that in addition to assessing their contribution to the downstream task ("Evaluation on downstream task: classification"), we also evaluate the quality of the synthesized samples, in terms of realism, diversity, and richness of labels [55, 56]. We now elaborate these properties in context of our work below.

### Realism and label correctness

While it is desirable that the output of any augmentation method be different from the original data in order to better minimize $R_{\textrm{vic}}$ ("Method"), we want to avoid sampling synthetic points off the original data manifold, thereby also ensuring trustworthy machine learning [90].

Consider the CRESCENTS and the SPIRALS datasets, two 2D synthetic data distribution described in "Synthetic Data" Section and visualized as “Input” in Fig. 2a. Applying *mixup*  to CRESCENTS and SPIRALS datasets shows that *mixup*  does not respect the individual class boundaries and synthesizes samples off the data manifold, also known as manifold intrusion [25]. This also results in the generated samples being wrongly labeled, i.e.,  points in the “red” class’s region being assigned “blue” labels and vice versa, which we term as “label error”. On the other hand, $\zeta$-*mixup*  preserves the class decision boundaries irrespective of the hyperparameter $\gamma$ and additionally allows for a controlled interpolation between the original distribution and *mixup*-like output. With $\zeta$-*mixup*, small values of $\gamma$ (greater than $\gamma _{\textrm{min}}$; see Theorem 1) lead to samples being generated further away from the original data and as $\gamma$ increases, the resulting distribution approaches the original data.

Applying *mixup*  in 3D space (Fig. 2b) results in a somewhat extreme case of the generated points sampled off the data manifold, filling up the entire hollow region in between the helical distribution. $\zeta$-*mixup*, however, similar to Fig. 2a, generates points that are relatively much closer to the original points, and increasing the value of $\gamma$ to a large value, say $\gamma =6.0$, leads the generated samples to lie almost perfectly on the original data manifold.

Moving on to higher dimensions with the MNIST data, i.e.,  784-D, we observe that the problems with *mixup* ’s output are even more severe and that the improvements by using $\zeta$-*mixup*  are more conspicuous. For each digit class in the MNIST dataset, we take the first 10 samples as shown in Fig. 3a and use *mixup*  and $\zeta$-*mixup*  to generate 100 new images each (Fig. 3b, c). It is easy to see that the digits in $\zeta$-*mixup* ’s output are more discernible than those in *mixup* ’s output.

Finally, to analyze the correctness of probabilistic labels in the outputs of *mixup*  and $\zeta$-*mixup*, we pick 4 samples each from the respective outputs and inspect their probabilistic soft labels. *mixup* ’s outputs (Fig. 3d) all look like images of handwritten “8”. The soft label of the first digit in Fig. 3d is [0, 0.53, 0, 0, 0, 0.47, 0, 0, 0, 0], where the $i^{\textrm{th}}$ index is the probability of the $i^{\textrm{th}}$ digit, implying that this output has been obtained by mixing images of digits “1” and “5”. Interestingly, neither the resulting output looks like the digits “1” or “5” nor is the digit “8” one of the classes used as input for this image. I.e., there is a disagreement, with *mixup*, between the appearance of the synthesized image and its assigned label. Similar label error exists in the other images in Fig. 3d. On the other hand, there is a clear agreement between the images produced by $\zeta$-*mixup*  and the labels assigned to them (Fig. 3e).

Next, we set out to quantify (i) realism and (ii) label correctness of *mixup*  and $\zeta$-*mixup*-synthesized images. To this end, we assume access to an Oracle that can recognize MNIST digits. For (i), we hypothesize that the more an image is realistic, the more the Oracle will be certain about the digit in it, and vice-versa. For example, although the first image in Fig. 3d is a combination of a “1” and a “5”, the resulting image looks very similar to a realistic handwritten “8”. On the other hand, consider the highlighted and zoomed digits in Fig. 3b. For an Oracle, images like these are ambiguous and do not belong to one particular class. Consequently, the uncertainty of the Oracle’s prediction will be high. We therefore adopt the Oracle’s entropy (${\mathcal {H}}$) as a proxy for realism. For (ii), we use cross entropy (CE) to compare the soft labels assigned by either *mixup*  or $\zeta$-*mixup*  to the label assigned by the Oracle. For example, if the resulting digit in a synthesized image is deemed an “8” to an Oracle and the label assigned to the sample, by *mixup*  or $\zeta$-*mixup*, is also “8”, then the CE is low and the label is correct. We also note that for the Oracle, the certainty of the predictions is correlated with the correctness of label. Finally, to address the issue of what Oracle to use, we adopt a highly accurate LeNet-5 [26] MNIST digit classifier that achieves $99.31\%$ classification accuracy on the standardized MNIST test set.

Figure 3f, g show the quantitative results for the realism ($\propto$ 1/${\mathcal {H}}$) of *mixup*  and $\zeta$-*mixup* ’s outputs, and the correctness of the corresponding labels ($\propto$ 1/CE) as evaluated by the Oracle, respectively, using kernel density estimate (KDE) plots with normalized areas. For both metrics, lower values (along the horizontal axes) are better. In Fig. 3f, we observe the $\zeta$-*mixup*  has a higher peak for low values of entropy as compared to *mixup*, indicating that the former generates more realistic samples. The inset figure therein shows the same plot with a logarithmic scale for the density, and $\zeta$-*mixup* ’s improvements over *mixup*  for higher values of entropy are clearly discernible here. Similarly, in Fig. 3g, we see that the cross entropy values for $\zeta$-*mixup*  are concentrated around 0, whereas those for *mixup*  are spread out more widely, implying that the former produces fewer samples with label error. If we restrict our samples to only those whose entropy of Oracle’s predictions was less than 0.1, meaning they were highly realistic samples, the label correctness distribution remains similar as shown in the inset figure, i.e.,  *mixup* ’s outputs that look realistic are more likely to exhibit label error.

Note that similar problems with unrealistic synthesized images exist with skin lesion images, as shown in the outputs of *mixup*  applied to 100 samples from ISIC 2017 (Fig. 4) and ISIC 2018 (Fig. 5) datasets. *mixup*  generates images that contain (1) overlapping lesions with different diagnoses, (2) overlapping artifacts (dark corners, stickers, ink markers, hair, etc.) overlapping the lesion, or (3) images with unrealistic anatomical arrangements such as lesion or hair appearing outside the body. However, despite $\zeta$-*mixup* ’s outputs exhibiting a higher degree of realism compared to those of *mixup*, we acknowledge that it is difficult to accurately estimate the realism of medical images without expert assessment.

### Diversity

We can control the diversity of $\zeta$-*mixup* ’s output by changing ${T}$, i.e.,  the number of points used as input to $\zeta$-*mixup*, and the hyperparameter $\gamma$. As the value of $\gamma$ increases, the resulting distribution of the sampled points approaches the original data distribution. For example, in Fig. 2a, we see that changing $\gamma$ leads to an interpolation between *mixup*-like and the original input-like distributions. Similarly, in Fig. 2c, we can see the effects of varying the batch size ${T}$ (i.e.,  the number of input samples used to synthesize new samples) and $\gamma$. As ${T}$ increases, more original samples are used to generate the synthetic samples, and therefore the synthesized samples allow for a wider exploration of the space around the original samples. This effect is more pronounced with smaller values of $\gamma$ because with the weight assigned to one point, while still dominating all other weights, is not large enough to pull the synthetic sample close to it. This, along with fewer points to compute the weighted average of, leads to samples being generated farther from the original distribution as $\gamma$ decreases. On the other hand, as $\gamma$ increases, the contribution of one sample gets progressively larger, and as a result, the effect of a large $\gamma$ overshadows the effect of ${T}$.

### Richness of labels

The third desirable property of synthetic data is that, not only the generated samples should be able to capture and reflect the diversity of the original dataset, but also build upon it and extend it. As discussed in "Method", for a single value of $\lambda$, *mixup*   generates 1 synthetic sample for every pair of original samples. In contrast, given a single value of $\gamma$ and ${T}$ original samples, $\zeta$-*mixup*  can generate ${T}!$ new samples. The richness of the generated labels in $\zeta$-*mixup*  comes from the fact that, unlike *mixup*  whose outputs lie anywhere on the straight line between the original 2 samples, $\zeta$-*mixup*  generates samples which are close to the original samples (as discussed in “Realism” above) while still incorporating information from the original ${T}$ samples. As a case in point, consider the visualization of the soft labels in *mixup* ’s and $\zeta$-*mixup* ’s outputs on the MNIST dataset. Examining Fig. 3b, d again, we note *mixup* ’s outputs are only made up of inputs from at most 2 classes. On the other hand, because of $\zeta$-*mixup* ’s formulation, the outputs of $\zeta$-*mixup*  can be made up of inputs from up to $min\left( {T}, {\mathcal {K}}\right)$ classes. This can also be seen in $\zeta$-*mixup* ’s outputs in Fig. 3e: while the probability of one class dominates all others (see Theorem 1), inputs from multiple classes, in addition to the dominant class, contribute to the final output and therefore this is reflected in the soft labels, leading to richer labels with information from multiple classes in 1 synthetic sample, which in turn arguably allow models trained on these samples to better learn the class decision boundaries.

### Preserving the intrinsic dimensionality of the original data

As a direct consequence of the realism of synthetic data discussed above and its relation to the data manifold, we evaluate how the intrinsic dimensionality (ID hereafter) of the datasets change when *mixup*  and $\zeta$-*mixup*  are applied.

According to the manifold hypothesis, the probability mass of high-dimensional data such as images, speech, text, etc. is highly concentrated, and optimization problems in such high dimensions can be solved by fitting low-dimensional non-linear manifolds to points from the original high-dimensional space, with this approach being known as manifold learning [53, 54, 59]. This idea that real world image datasets can be described by considerably fewer dimensional representations [91], also known as the intrinsic dimensionality, has fuelled research into lower dimensional representation learning techniques such as autoencoders [92, 93]. Moreover, recent research has concluded that deep learning models are easier to train on datasets with low dimensionalities and that such models exhibit better generalization performance [45].

While the ID of a dataset can be estimated globally, datasets can have heterogeneous regions and thus consist of regions of varying IDs. As such, instead of a global estimate of the ID, a local measure of the ID (local ID hereafter), estimated in the local neighborhood of each point in the dataset with neighborhoods typically defined using the *k*-nearest neighbors, is more informative of the inherent organization of the dataset. For our local ID estimation experiments, we use a principal component analysis-based local ID estimator from the scikit-dimension Python library [94] using the Fukunaga-Olsen method [95], where an eigenvalue is considered significant if it is larger than $5\%$ of the largest eigenvalue.

With our 3D manifold visualizations in Fig. 2b, we saw that *mixup*  samples points off the data manifold while $\zeta$-*mixup*  limits the exploration of the high-dimensional space, thus maintaining a lower ID. In order to substantiate this claim with quantitative results, we estimate the IDs of several datasets, both synthetic and real-world, and compare how the IDs of *mixup*- and $\zeta$-*mixup*-generated distributions compare to those of the respective original distributions. For synthetic data, we use the high-dimensional datasets described in "Synthetic data", i.e.,  1-D helical manifolds embedded in $\mathbb {R}^3$ and in $\mathbb {R}^{12}$. For real-world datasets, we use the entire training partitions (50,000 images) of CIFAR-10 and CIFAR-100 datasets.

For each point in all the 4 datasets, the local ID is calculated using a *k*-nearest neighborhood around each point with $k=8$ and $k=128$ [94, 95]. The means and the standard deviations of the local ID estimates for all the datasets: original data distribution, *mixup* ’s output, and $\zeta$-*mixup* ’s outputs for $\gamma \in [0, 15]$, are visualized in Fig. 6.

The results in Fig. 6 support the observations from the discussion around the realism ("Realism and Label Correctness" Section) and the diversity ("Diversity") of outputs. In particular, notice how *mixup* ’s off-manifold sampling leads to an inflated estimate of the local ID, whereas the local ID of $\zeta$-*mixup* ’s output is lower than that of *mixup*  and, as expected, can be controlled using $\gamma$. This difference is even more apparent with real-world high-dimensional (3072-D) datasets, i.e.,  CIFAR-10 and CIFAR-100, where for all values of $\gamma \ge \gamma _{\textrm{min}}$ (Theorem 1), as $\gamma$ increases, the local ID of $\zeta$-*mixup* ’s output drops dramatically, meaning the resulting distributions lie on progressively lower dimensional intrinsic manifolds.

We note, however, that for some datasets,when employing large values of $\gamma$, the local ID of $\zeta$-*mixup* outputs may be lower than the local ID of the original dataset (Fig. 6). Since we use the same number of nearest neighbors ($n_{\textrm{NN}} = \{8, 128\}$) across all methods to perform PCA-based local ID estimation [95], higher values of $\gamma$ lead to synthesized samples being closer to each other and the distribution of the resulting augmented samples being more compact than the original dataset (“vanilla” in Fig. 6). Fig. 7 shows a visual explanation for this: consider a synthetic two-class 2D data distribution, and its *mixup* and $\zeta$-*mixup* augmented outputs (Fig. 7a–c) respectively). We see that if we were to estimate the local ID for this data without any augmentation (Fig. 7d), the samples are comparatively more spread out, compared to $\zeta$-*mixup* outputs (Fig. 7e). If we were to fit an ellipse (representing the covariance of the data or the result of PCA) to estimate the local ID, notice how $\zeta$-*mixup* ’s more compact distribution leads to an ellipse with higher eccentricity than the one for the original distribution.

### Evaluation on downstream task: classification

We compare the classification performance of models trained using traditional data augmentation techniques, e.g.,  rotation, horizontal and vertical flipping, and cropping (“ERM”), against those trained with *mixup* ’s and $\zeta$-*mixup* ’s outputs. Additionally, we also evaluate if there are performance improvements when $\zeta$-*mixup*  is applied in conjunction with an orthogonal augmentation technique, CutMix.

We do not compare against optimization-based mixing methods (e.g.,  Co-Mixup [96]), which, while conceptually orthogonal to $\zeta$-*mixup* and potentially complementary, involve the use of combinatorial optimization and specialized libraries^1^. These methods, by design, introduce a significant computational overhead that places the burden of image understanding on the data augmentation process. This increased computational cost is evident in model training times. For instance, CIFAR-100 models trained using *mixup*, $\zeta$-*mixup*, CutMix, and even the combination of CutMix and $\zeta$-*mixup* take up almost the same time as ERM (approximately 1h 20 m; Table 9). On the other hand, Co-Mixup, due to its reliance on optimation, requires training times that are over an order of magnitude larger (over 16h; similar to the training time in the official repository’s training log^2^). We also refrain from extensive comparison against methods that interpolate in the latent space (e.g.,  *manifold mixup* [41]) for two main reasons. First, the the computational demands associated with these methods are considerably higher: while ERM, *mixup*, $\zeta$-*mixup* models trained on CIFAR-100 converge in a reasonable amount of time, typically within 200 epochs and approximately 1 h, training a model with *manifold mixup* extends to 2000 epochs and requiring over 16  h (Table 9). Moreover, the theoretical justifications associated with such methods are not unanimously agreed upon [97]. Nevertheless, despite this considerably higher computational burden, we compare *manifold mixup* to $\zeta$-*mixup* on nine diverse natural and medical image classification datasets.

Table 4 presents the quantitative evaluation for the natural image datasets. For all our experiments with *mixup*, we use the official implementation by the authors^3^. *mixup* samples its interpolation factor $\lambda$ from a Beta($\alpha , \alpha$) distribution, and following the original *mixup* paper [36], their code implementation^4^, as well as several other works [39, 42, 44, 98–100], we set $\alpha = 1$, which results in $\lambda$ being sampled from a $\textrm{U}[0, 1]$ uniform distribution. For all our experiments with $\zeta$-*mixup*, we synthesize new training samples through convex combinations (Eqn. 5, Eqn. 6) of all the samples in a training batch, i.e., *T* (number of samples used for interpolation) $= m$ (number of samples in a training batch). For comparison against *mixup*-based models, we choose 3 values of $\gamma$ for the corresponding $\zeta$-*mixup*-based models:

- $\gamma =2.4$: to allow exploration of the space around the original data manifold,
- $\gamma =4.0$: to restrict the synthetic samples to be close to the original samples, and
- $\gamma =2.8$: to allow for a behavior that permits exploration while still restricting the points to a small region around the original distribution.

We see that 17 of the 18 models in Table 4 trained with $\zeta$-*mixup*  outperform their ERM and *mixup*  counterparts, with the lone exception being a model that is as accurate as *mixup*. We also observe a performance improvement when $\zeta$-*mixup*  is applied along with CutMix, as shown in Table 5. To show that the performance gains from $\zeta$-*mixup*  are achievable for all reasonable values of $\gamma$, for these experiments, we sample a new $\gamma \in \textrm{U}[1.72865, 4.0]$ for each mini-batch.

Next, Table 6 shows the performance of the models on the 10 skin lesion image diagnosis datasets ($\gamma =\{2.4, 2.8, 4.0\}$). For both ResNet-18 and ResNet-50 and for all the 10 SKIN datasets, $\zeta$-*mixup*  outperforms both *mixup*  and ERM on skin lesion diagnosis tasks. Finally, Table 7 presents the quantitative evaluation on the 8 classification datasets from the MedMNIST collection, but use $\zeta$-*mixup*  only with $\gamma =2.8$. In 8 out of the 10 datasets, $\zeta$-*mixup*  outperforms both *mixup*  and ERM, and in the other 2, $\zeta$-*mixup*  achieves the highest value for 1 metric out of 2 each.

Note that these selected values of $\gamma$ can be changed to other reasonable values (see "[Sec Sec20]" for sensitivity analysis of $\gamma$), and as shown above qualitatively and quantitatively, the desirable properties of $\zeta$-*mixup*  hold for all values of $\gamma \ge \gamma _{\textrm{min}}$. Consequently, our quantitative results on classification tasks on 26 datasets show that $\zeta$-*mixup*  outperforms ERM and *mixup*  for all the datasets and, in most cases, using all three selected values of $\gamma$.

For a more intuitive explanation of how $\zeta$-*mixup* leads to superior performance, let us revisit the synthetic data distribution in Fig. 7, now with a test sample (denoted by a green square). With *mixup*, the test sample may lie in the vicinity of incorrectly labeled *mixup*-augmented training samples. We study the classes of the samples in the vicinity of a test sample using its *k*-nearest neighbors, $k = \{8, 16\}$. Such errors, i.e., a test sample falling in the vicinity of training samples of a different class leading to misclassification, are less likely with $\zeta$-*mixup* since it generates training samples that are closer to the original data distribution.

This can also be observed on real-world datasets. We choose two skin lesion image datasets from our experiments spanning two imaging modalities, and two model architectures for our analysis: the ResNet-50 model trained on ISIC 2017 (dermoscopic images) and the ResNet-18 model trained on derm7point: Clinical (clinical images). Fig. 8a shows 14 sample images from the test sets of each of the two datasets that were misclassified by both ERM and *mixup*, but were correctly classified by $\zeta$-*mixup* for all values of $\gamma$ (Table 6). To study the distribution of training samples and their labels in the vicinity of these test images, we perform the following analysis: for both the models, we generate *mixup*- and $\zeta$-*mixup*-synthesized training samples, and compute their features using the pre-trained classification models. This results in 2048-dimensional and 512-dimensional feature vectors for ISIC 2017 (ResNet-50) and derm7point (ResNet-18), respectively. For 12 of these 14 test images from derm7point (Fig. 8a), there were more training samples with correct labels in the vicinity of the test samples (measured by calculating the 128-nearest neighbors in the 512-dimensional feature space) for the $\zeta$-*mixup*-trained model than the *mixup*-trained model. Overall, the number of correctly labeled nearest neighbor training samples was $208.2\%$ more for $\zeta$-*mixup* compared to *mixup*. The corresponding numbers for ISIC 2017 (2048-dimensional feature space) were 14 out of 14 test samples and $1908.8\%$ more correctly labeled nearest neighbor training samples. The distances for the nearest neighbors were calculated using cosine similarity.

Next, we project these onto a 2D embedding space through t-distributed Stochastic Neighbor Embedding (t-SNE) [101] using the openTSNE Python library [102], representing each training sample’s feature using a class color-coded circle. Finally, we project the test samples’ features onto the same embedding spaces, denoted by squares. It should be noted that this t-SNE representation drastically reduces the dimensionality of the features ($\{512, 2048\}$-D $\rightarrow 2$-D), causing some information loss. We observe that with *mixup* (Fig. 8b, d), several test samples fall in the vicinity of training samples of a different class than the correct class of the test sample, potentially leading to misclassification. Examples of this include a ‘NEV’ misclassified as ‘MEL’, ‘NEV’ misclassified as ‘SK’, and ‘SK’ misclassified as ‘NEV’ in Fig. 8b and ‘NEV’ misclassified as ‘MEL’ and ‘MISC’ misclassified as ‘MEL’ in Fig. 8d. With $\zeta$-*mixup*, on the other hand, these test samples are less likely to have training images of a different class than the test sample’s class in their vicinity (Fig. 8c, e).

Finally, we also compare $\zeta$-*mixup* to the computationally intensive *manifold mixup*. As mentioned above, *manifold mixup* requires an order of magnitude more number of epochs for convergence. For instance, while all of ERM, *mixup*, and $\zeta$-*mixup* require 200 epochs, $\zeta$-*mixup* is trained for 2000 epochs [41]. However, in an effort to understand the performance gains obtained from such a massive computational requirement, we evaluate *manifold mixup* on 9 datasets: we choose 2 datasets from NATURAL (CIFAR-10, CIFAR-100), 3 datasets from MEDMNIST (BreastMNIST, PathMNIST, TissueMNIST), and 4 datasets from SKIN (derm7point: Clinical, MSK, ISIC 2017, DermoFit), thus covering natural and medical image datasets of various resolutions ($28 \times 28$, $32 \times 32$, $224 \times 224$), multiple medical imaging modalities (dermoscopic and clinical skin images, ultrasound images, histopathology images, microscopic images), image types (BreastMNIST and TissueMNIST are grayscale while others are RGB), and model architectures (ResNet-18, ResNet-50). For CIFAR-10 and CIFAR-100, we follow the experimental settings of Verma et al. [41], and since they did not perform experiments on our other datasets, we scale the corresponding experimental settings (i.e., the number of training epochs and the learning rate scheduler milestones) accordingly. Therefore, for the 3 MEDMNIST datasets, the *manifold mixup*-augmented classification models are trained for 1, 000 epochs with a learning rate of 0.01. For the 4 SKIN datasets, the *manifold mixup* models are trained for 500 epochs with an initial learning rate of 0.01 decayed by a multiplicative factor of 0.1 every 100 epochs. The quantitative results for all metrics in all datasets are visualized in Fig. 9. For 2 datasets, *manifold mixup* outperforms $\zeta$-*mixup*, and for 3 datasets, *manifold mixup* achieves one superior metric than $\zeta$-*mixup*. However, for 4 datasets, $\zeta$-*mixup* outperforms *manifold mixup* across all metrics. Therefore, despite being considerably more computationally intensive (each *manifold mixup* model is trained for $10\times$ the number of epochs compared to a $\zeta$-*mixup* trained on the same dataset), *manifold mixup*-trained models do not demonstrate a clear and consistent performance improvement over the comparatively more efficient $\zeta$-*mixup*.

### $\zeta$-*mixup*: hyperparameter sensitivity analysis and ablation study

We conduct extensive experiments on CIFAR-10 and CIFAR-100 datasets to analyze the effect of $\zeta$-*mixup* ’s hyperparameter: $\gamma$ on the performance of $\zeta$-*mixup*, and also analyze how the weight-decay of SGD-based optimization affects model performance.

First, we vary the hyperparameter $\gamma$ by choosing values from [1.8, 2.0, 2.2, $\cdots$, 5.0] and train and evaluate ResNet-18 models on CIFAR-10 and CIFAR-100. The corresponding overall error rates (ERR) are shown in Fig. 10 (a) and (b), respectively. We observe that for almost all values of $\gamma$, $\zeta$-*mixup* achieves lower or equal error rate (ERR) than *mixup*, thus supporting our claims with our results on 26 datasets that performance gains with $\zeta$-*mixup* are achievable for all values of $\gamma \ge \gamma _{\textrm{min}}$.

To further understand the effect of $\zeta$-*mixup* augmentation on model optimization in the presence of weight decay, we perform another extensive hyperparameter study: we observe model performance by varying both $\gamma$ and the weight decay ($L_2$ penalty) for SGD. We sample the hyperparameter $\gamma$ from a uniform distribution over [1.0, 6.0] and the weight decay from a log-uniform distribution over $[5e-5, 1e-3]$, and use Weights and Biases [103] to perform a Bayesian search [104–107] in this space. We train and evaluate ResNet-18 models on the CIFAR-10 and CIFAR-100 datasets. For each of the two datasets, we train 200 models, each optimized with a different combination of $\gamma$ and weight decay. To visualize the results, we plot three values: $\gamma$, weight decay, and final test accuracy of the resultant model using parallel coordinates plots [108, 109] (Fig. 10c, d). Models trained with $\gamma < \gamma _{\textrm{min}}$ are shown in light gray.

The parallel coordinates plots can be read by following a curve through the 3 columns, where each curve denotes an experiment with the values of, in order left-to-right, $\gamma$, weight decay, and test accuracy. For all columns, a lighter color indicates a higher value. We observe that the best performing models (i.e., the curves with the lightest shades of yellow) emanate from smaller values of $\gamma$ (i.e., approximately in the range of $[1.72865, 4.0]$) and larger weight decays (approximately in the range of $[5e-4, 1e-3]$). On the other hand, larger values of $\gamma$, which lead to data distributions similar to the “vanilla” distribution (Fig. 2a), yield lower classification accuracies (i.e., the curves with dark purple colors), validating our hypothesis that the augmented samples do not considerably explore the space around the original samples.

Finally, to understand the individual contribution of each of the two components of $\zeta$-*mixup*: the mixing of all the samples in a batch (i.e., $T=m$ original samples; Eq. 5) and sampling of weights from a normalized *p*-series for the original samples (Eq. 6), towards its superior performance, we perform the following ablation study. We train models with one of these components removed at a time, and study the effect on the downstream classification performance. For this, we use the CIFAR-100 dataset because of its large number of classes (100) and use the experimental settings from "Evaluation on downstream task: classification" and Table 4: ResNet-18 architecture trained for 200 epochs with an initial learning rate of 0.1 decayed by a multiplicative factor of 0.2 at 80, 120, and 160 epochs, $\gamma = 2.8$, and $m=128$. The quantitative results for this ablation study are presented in Table 8. To begin with, note that *mixup* is a special case of $\zeta$-*mixup* (Theorem 2) where the former uses neither of the aforementioned components. Then, we modify *mixup* to mix samples using the proposed weighting scheme (Eq. 6) while retaining *mixup* ’s choice of mixing only 2 samples. This results in an improved performance over *mixup*. For the next experiment, we mix the entire batch (i.e., $T=m$) but with weights sampled from a Dirichlet distribution $\textrm{Dir} (\varvec{\alpha })$ with $\varvec{\alpha } = [1.0, 1.0, \cdots 1.0]$, since this is a multivariate generalization of the Beta(1.0, 1.0) distribution-sampled weights used for *mixup*. Unsurprisingly, we observe that mixing a large number of samples ($m=128$) with a weighting scheme that does not have a large weight assigned to a single sample results in very poor performance. Such a weighting scheme violates one of the desirable properties of an ideal augmentation method ("[Sec Sec5]"), since the synthesized samples will be generated away from the original samples, leaving the original data manifold (Fig. 1) and therefore exhibit a higher local intrinsic dimensionality (Fig. 6) and lower realism. Finally, $\zeta$-*mixup*, which uses both of these components, outperforms all these methods.

### Computational efficiency

The $\zeta$-*mixup*  implementation in PyTorch [110] is shown in Listing 1. Unlike *mixup*  which performs scalar multiplications of $\lambda$ and $1-\lambda$ with the input batches, $\zeta$-*mixup*  performs a single matrix multiplication of the input batches with the weights. With our optimized implementation, we find that model training times using $\zeta$-*mixup*  are as fast as, if not faster than, those using *mixup*  when evaluated on datasets with different spatial resolutions: CIFAR-10 ($32 \times 32$ RGB images), STL-10 ($96 \times 96$ RGB images), and Imagenette ($224 \times 224$ RGB images), as shown in Table 9. Moreover, when using *mixup*  and $\zeta$-*mixup*  on a batch of 32 tensors of $224 \times 224$ spatial resolution with 3 feature channels, which is the case with popular ImageNet-like training regimes, $\zeta$-*mixup*  is over twice as fast as *mixup*  and over 110 times faster than the original local synthetic instances implementation [57].

## Conclusion

We proposed $\zeta$-*mixup*, a parameter-free multi-sample generalization of the popular *mixup*  technique for data augmentation that uses the terms of a truncated Riemann zeta function to combine ${T}\ge 2$ samples of the original dataset without significant computational overhead. We presented theoretical proofs that *mixup*  is a special case of $\zeta$-*mixup*  (when ${T}=2$ and with a specific setting of $\zeta$-*mixup* ’s hyperparameter $\gamma$) and that the $\zeta$-*mixup*  formulation allows for the weight assigned to one sample to dominate all the others, thus ensuring the synthesized samples are on or close to the original data manifold. The latter property leads to generating samples that are more realistic and, along with allowing ${T} > 2$, generates more diverse samples with richer labels as compared to their *mixup*  counterparts. We presented extensive experimental evaluation on controlled synthetic (1-D manifolds in 2-D and 3-D; 3-D manifolds in 12-D) and 26 real-world (natural and medical) image datasets of various modalities. We demonstrated quantitatively that, compared to *mixup*: $\zeta$-*mixup*  better preserves the intrinsic dimensionality of the original datasets; provides higher levels of realism and label correctness; and achieves stronger performance (i.e., higher accuracy) on multiple downstream classification tasks. Future work will include exploring $\zeta$-*mixup*  in the learned feature space, although opinions on the theoretical justifications for interpolating in the latent space are not yet converged [97].

**Algorithm 1:**
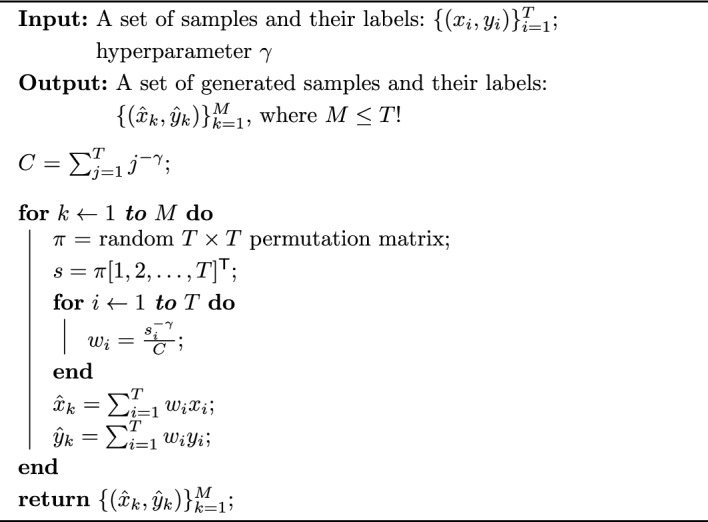
$$\zeta $$-*mixup*


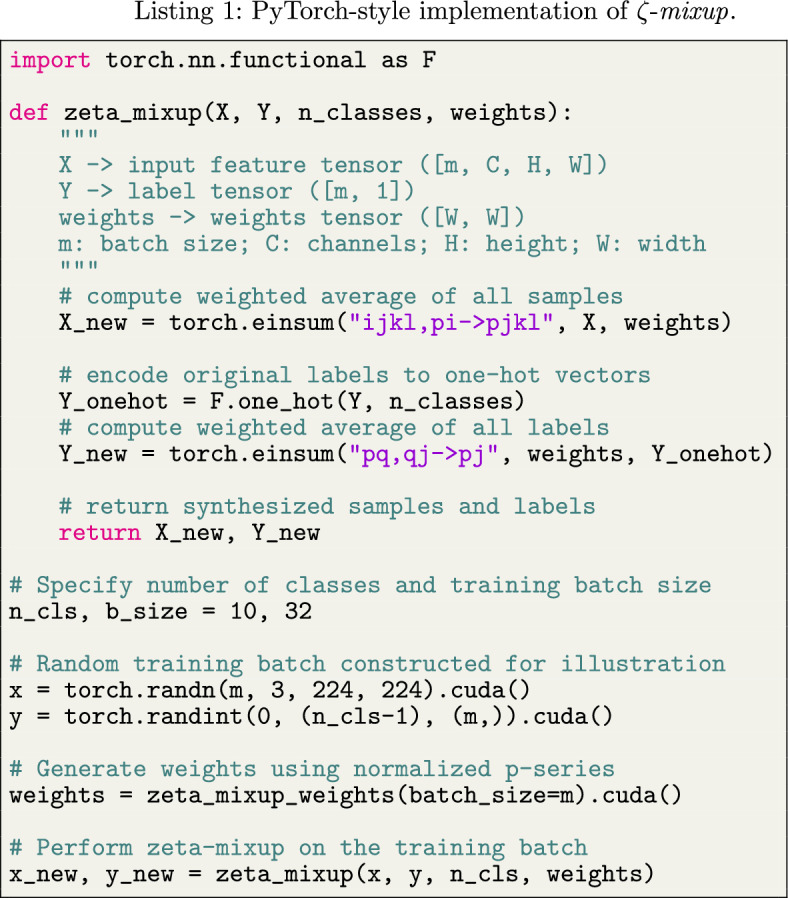


## Data Availability

All the datasets used in this research, except DermoFit [76], are publicly available and can be downloaded from their respective websites. DermoFit [76] is available through an academic license from the University of Edinburgh. The download links for all the datasets are listed below: MNIST [26]: http://yann.lecun.com/exdb/mnist/. CIFAR-10 and CIFAR-100 [63]: https://www.cs.toronto.edu/~kriz/cifar.html. Fashion-MNIST [64]: https://www.github.com/zalandoresearch/fashion-mnist. STL-10 [65]: https://cs.stanford.edu/~acoates/stl10/. Imagenette and Imagewoof [66]: https://www.github.com/fastai/imagenette. ISIC 2016 [71]: https://challenge.isic-archive.com/data/#2016. ISIC 2017 [72]: https://challenge.isic-archive.com/data/#2017. ISIC 2018 [73, 74]: https://challenge.isic-archive.com/data/#2018. MSK and UDA [75]: https://www.isic-archive.com/#!/topWithHeader/onlyHeaderTop/gallery. DermoFit [76]: https://licensing.edinburgh-innovations.ed.ac.uk/product/dermofit-image-library. derm7point [77]: https://derm.cs.sfu.ca/. PH2 [78]: https://www.fc.up.pt/addi/ph2%20database.html. MED-NODE [79]: https://www.cs.rug.nl/~imaging/databases/melanoma_naevi/. MedMNIST [83]: https://www.medmnist.com/

## Abbreviations

DNN: Deep neural network
ERM: Empirical risk minimization
VRM: Vicinal risk minimization
F-MNIST: Fashion-MNIST
SGD: Stochastic gradient descent
ISIC: International Skin Imaging Collaboration
MSK: Memorial Sloan-Kettering
CE: Cross entropy
KDE: Kernel density estimate
ID: Intrinsic dimensionality

## Footnotes

1 https://github.com/Borda/pyGCO.

2 https://github.com/snu-mllab/Co-Mixup/blob/main/checkpoint/cifar100_preactresnet18_eph300_comixup/log.txt.

3 https://github.com/facebookresearch/mixup-cifar10.

4 https://github.com/facebookresearch/mixup-cifar10/blob/main/train.py#L119.
